# Quality of life improved for patients after starting dialysis but is impaired, initially, for their partners: a multi-centre, longitudinal study

**DOI:** 10.1186/s12882-020-01819-4

**Published:** 2020-05-18

**Authors:** Currie Moore, Lesley-Anne Carter, Sandip Mitra, Suzanne Skevington, Alison Wearden

**Affiliations:** 1 School of Health Sciences, Division of Psychology and Mental Health, Manchester Centre for Health Psychology, University of Manchester, Manchester, UK; 2 Manchester Academic Health Science Centre, University of Manchester, Manchester, UK; 3 Division of Population Health, Health Services Research & Primary Care, University of Manchester, Manchester, UK; 4 Manchester University NHS Foundation Trust, Manchester, UK; 5 NIHR Devices for Dignity MedTech Cooperative, Sheffield, UK

**Keywords:** Quality of life, Dialysis, Caregiver, End stage renal disease, Multilevel model, Dyad

## Abstract

**Background:**

Quality of life (QOL) is important to patients with end stage renal disease and their partners. Despite the first 12 weeks being a critical time in the treatment pathway, limited research exists which examines how the transition onto dialysis impacts QOL. In this study we measured QOL in patients and their partners at pre-dialysis and over the first 12 weeks on dialysis to investigate QOL during this crucial period.

**Methods:**

Patients and their partners, recruited from 10 renal units in England, completed questionnaires at pre-dialysis (*n* = 166 participants, 83 couples), 6 weeks (*n* = 90 participants, 45 couples) and 12 weeks (*n* = 78, 39 couples) after starting dialysis. On each occasion participants completed a QOL questionnaire (WHOQOL-BREF). Multilevel modelling accommodated the nested structure of couples with repeated measures within participants. Three-level random intercept models estimated changes in WHOQOL general QOL and its four domains (Physical, Psychological, Social and Environment). Two-level random intercept models assessed the relationship between baseline clinical and socio-demographic variables with changes in general QOL.

**Results:**

Patients reported positive changes in general QOL from pre-dialysis to 6 weeks (β = 0.42, *p* < 0.001, 95% CI 0.19, 0.65) and from pre-dialysis to 12 weeks (β = 0.47, *p* < 0.001, 95% CI 0.24, 0.71). Partners’ general QOL decreased significantly from pre-dialysis to 6 weeks (β = − 0.24, *p* = 0.04, 95% CI -0.47, − 0.01) but returned to its original level at 12 weeks. Patients reported improvements in the physical domain between pre-dialysis and 12 weeks (β = 6.56, *p* < 0.004, 95% CI 2.10, 11.03). No other domains changed significantly in patients or partners. Only in patients were there significant associations between moderator variables and general QOL. High comorbidity risk level and diabetes were associated with poorer QOL at pre-dialysis whereas being female and having an arteriovenous fistula were linked with improvements in general QOL.

**Conclusions:**

Patients reported significant improvements in their general and physical QOL after starting dialysis. Partners’ general QOL worsened after patients started dialysis but improved by 12 weeks. Both patients and partners may benefit from additional educational and counselling services in the lead up to, and immediately after starting dialysis, which could facilitate the transition onto dialysis and improve QOL in both.

**Study registration:**

This study was adopted on the NIHR Clinical Research Network (UK). The details of this study are registered on the Research Registry website (www.researchregistry.com). The identifier for this study is researchregistry2574.

## Background

When preparing to start dialysis, patients with end stage renal disease (ESRD) and their primary caregivers (partners) consistently state that quality of life (QOL) is an important concern [1–3]. Quality of life (QOL) is defined by the World Health Organization [4] as “*an individual’s perception of their position in life in the context of the culture and value systems in which they live, and in relation to their goals, expectations, standards and concerns*” (p. 43). This definition complements the ESRD illness trajectory of dialysis patients, as described by Jablonski [5], which indicates that changes occur in all areas of patients’ lives as they start and initially adjust to dialysis, referred to as the “crisis” phase (p. 54). Psychosocial interventions delivered in the critical first 12 weeks have been linked with better adjustment and decreased levels of depression in patients [6]. Despite general well-being linked to better compliance, attendance of dialysis sessions and adjustment [7], research primarily focuses on patient survival or hospitalization rates rather than examining QOL [8, 9]. Although playing a key role in patients’ dialysis care [10, 11], little is known about how partners’ QOL is affected during the crisis phase.

Quality of life is a broad field with a multitude of scales to measure QOL and factors relating to it. Although patients and partners state QOL is a primary concern, it is not included as a core outcome measure in ESRD randomized control trials due to a lack of consensus on how best to define and measurement it [12]. Therefore, ESRD research may benefit from using a generic QOL measure which directly addresses QOL, rather than ESRD-specific or health status measures, and assesses several areas related to QOL (e.g., physical and emotional health, social support). One such measure is the World Health Organization’s WHOQOL-BREF short questionnaire [13] which was created by an international collaboration using an inductively-driven process, with the specific aim of designing a scale to measure QOL across all types of chronic illnesses and healthy people [14]. It includes a general QOL facet covering overall QOL and health and four QOL domains, physical, psychological, social and environment. Furthermore, patients who use haemodialysis (HD) and peritoneal dialysis (PD) and their partners have outlined energy, fatigue, ability to travel, work, and sleep [15, 16] as outcomes important to them, all of which are assessed in the WHOQOL-BREF.

Qualitative research including patients with ESRD and their partners suggests that they experience significant lifestyle changes as they adjust to dialysis [17–19] which impacts important areas of their QOL [20]. Our recent research with patients in the early phases of dialysis (i.e., pre-dialysis or on dialysis < 15 months) and their partners found that couples who adopted a team-like approach minimized the impact of dialysis on their relationship, and ultimately their QOL [17]. The findings of this study also suggest that starting dialysis affects patients and partners differently. For example, patients spoke of dialysis alleviating their worries and improving their health whereas partners reported an increase in burdens and significant changes in their personal lives.

To date, only one longitudinal study has examined how health status in both patients and partners changes after the start of dialysis. Fan et al. [21] conducted a single-centre study with patients on PD and their partners. Both completed the Medical Outcomes Study 36-Item Short Form Survey (SF-36), which measures various components of physical (PCS) and mental functioning (MCS), before the patient started PD and then again 1 year later. Before starting PD, PCS and MCS scores for both were lower than those of the general population. At 1 year, PCS and MCS scores improved modestly, and the social functioning subscale of the MCS showed statistically significant improvements in patients and partners. It remains unclear how starting dialysis impacts patients and their partners on other types of dialysis (e.g., HD). Further research is needed to examine the critical first 12 weeks and investigate QOL in its broader conceptualisation, rather than focusing on functioning as measured by the SF-36.

The aim of this study was to assess changes in QOL in patients and their partners, measured by the WHOQOL-BREF, during the crucial transition onto dialysis, from pre-dialysis to 12 weeks after starting dialysis. The primary goal was to describe QOL changes in WHOQOL general QOL and its domains over this transition period, in both patients and their partners. We also sought to examine whether starting dialysis affects patients’ and their partners’ QOL differently. We predicted that patients would report improvements in general QOL at 12 weeks, and their partners’ QOL would steadily worsen. Additionally, we examined whether clinical and socio-demographic factors were associated with changes in QOL.

## Methods

### Study design and setting

We used a multi-centre, longitudinal design to study changes in QOL in couples in which one person was starting dialysis. To closely examine the transition onto dialysis, both members of the couple - the patient and their partner - completed the study measures on three occasions: baseline (pre-dialysis) and two follow-ups (6 weeks and 12 weeks after starting dialysis). The study was adopted onto the National Institute for Health Research (NIHR) Clinical Research Portfolio (CPMS ID 35781) and conducted with 10 renal units across England.

### Study participants

Participants were recruited from November 2017–September 2018. Renal research nurses or members of the clinical care teams identified potential participants from their renal registries. Patients meeting the following criteria were eligible to participate: 1) they were in the care of a nephrologist for ESRD, including patients who were starting dialysis in an unplanned manner (i.e., people who had been under the care of nephrologists for less than 3 months before commencing dialysis), 2) their clinical factors indicated that they were likely to start dialysis in the next 2 months (i.e., their clinical care team assessed the longitudinal trend of their estimated glomerular filtration rate [eGFR, an indicator of how well the kidneys are eliminating toxins; calculated using the CKD EPI equation in the UK], symptom burden, serum albumin, haemoglobin, and patient preference, in accordance with the UK’s NICE guidance on dialysis initiation), 3) they were planning to receive a form of out-patient dialysis for the management of ESRD, including all forms of HD and PD, 4) they were in a spousal-type relationship with someone they considered their “partner,” and 5) they were 18 years or older. Incident patients (i.e., those who had not been on a form of renal replacement therapy before) and patients who had a failing transplant and were planning to start dialysis, but had not been on any form of out-patient dialysis in the last 6 months, were included. Patients were not eligible for the study if they had acute kidney injuries or were receiving long-term inpatient dialysis for other health conditions.

A partner was defined as a person in a spousal-type relationship who provided informal care in the form of emotional, physical and/or treatment-related support to an eligible patient [22]. Partners either self-identified as the patient’s partner or were identified as such by the patient. Inclusion criteria for partners were that they were in a spousal-type relationship (e.g., spouse, partner, boyfriend, or girlfriend) with the patient and were 18 years or older. Both patients and partners needed to be able to read and comprehend English.

### Procedure

Those meeting the inclusion criteria were invited to participate by the site investigator (SI). If the SI was not part of the patient’s clinical care team, a member of the clinical care team obtained the patient’s consent to contact before the SI approached the patient. The SI gave potential participants a letter of invitation and information sheet and provided further information about the study. Participation in the study was voluntary, and all recruited participants gave their written informed consent prior to taking part. This study only included couples in which *both* patients and their partners consented to participate and where both returned baseline questionnaires. At the follow-ups, all data from patients and partners were included, even if one member of the couple did not complete the questionnaire.

Before administering the questionnaires, the SI reminded participants that the questions ask about their QOL, health and their thoughts about dialysis and provided them with paper-versions of the questionnaires, either directly at a clinical appointment or via the post. Patients and partners completed similar versions of the questionnaires at each assessment and were requested not to confer. Patients and partners completed the questionnaires at home (95%) or in the renal unit (5%). Only three (2%) requested the SI read the questionnaire aloud; SIs were trained to administer the questionnaires in a standard manner.

### Measures

Quality of life was assessed using the WHOQOL-BREF [13]. This instrument reflects a multi-dimensional model of subjective QOL in health and is assessed by 26 questions. Two questions (items) form the WHOQOL general QOL facet and 24 specific items are scored in one of four domains: physical, psychological, social relationship, and environmental QOL. Participants assess their QOL over the last 2 weeks and rate responses to each item on a five-point Likert interval scale, where higher scores indicate better QOL. The primary outcome variable in the present study was WHOQOL general QOL, which is the mean of the overall QOL item (How would you rate your quality of life? 1 = Very poor, 2 = Poor, 3 = Neither good nor poor, 4 = Good, 5 = Very good) and the health-related QOL item (How satisfied are you with your health? 1 = Very dissatisfied, 2 = Dissatisfied, 3 = Neither satisfied or dissatisfied, 4 = Satisfied, 5 = Very satisfied) with a scoring range of 1–5. A score of 3 indicates moderate QOL, or QOL that is okay. A score less than 3 is commonly regarded as indicating poor or very poor QOL. Scores greater than 3 indicate good to very good QOL. The WHOQOL-BREF domain scores were transformed onto a scale from 0 to 100 to facilitate comparisons between different domains with unequal item numbers. Domain scores less than 50 indicate poor or very poor QOL. This instrument has been validated in both well [14] and dialysis populations [23] and shows good internal consistency and construct validity [13]. Furthermore, it has been found to be fairly sensitive to change across health conditions [24, 25].

Additionally, participants completed questionnaires on key factors related to QOL, namely dialysis expectations, accepting dialysis, patient-partner relationship characteristics, anxiety, depression, and symptoms. Descriptive statistics of these variables are included in Table 1 but will not be discussed further.

**Table 1:** Baseline characteristics of participants with comparisons between those who did and did not complete the study

	*Patients*				*Partners*			
	Overall *N* = 83	Pre-dialysis only *n* = 45	Completed study *n* = 39	*p-*value	Overall N = 83	Pre-dialysis only n = 45	Completed study n = 39	*p-*value
*Socio-demographic characteristics*								
Male n (%)^	52 (63%)	26 (59%)	26 (67%)	0.51	31 (37%)	18 (41%)	13 (33%)	0.51
Age *M* (*SD*, years)	64 (14)	64 (14)	64 (14)	0.82	63 (15)	64 (15)	62 (15)	0.72
Married n (%)^	69 (84%)	36 (82%)	33 (87%)	5.29	70 (84%)	36 (82%)	34 (87%)	6.61
Highest level of education n (%)^				3.68				1.51
None	4 (5%)	3 (7%)	1 (3%)		4 (5%)	3 (7%)	1 (3%)	
Primary school	3 (4%)	2 (5%)	1 (3%)		2 (2%)	1 (2%)	1 (3%)	
Secondary school	40 (48%)	19 (43%)	21 (55%)		33 (40%)	18 (41%)	15 (38%)	
College or training certification	25 (30%)	15 (34%)	10 (26%)		36 (43%)	18 (41%)	18 (46%)	
University – undergraduate	4 (5%)	3 (7%)	1 (3%)		5 (6%)	3 (7%)	2 (5%)	
University – postgraduate	6 (7%)	2 (5%)	4 (11%)		3 (4%)	1 (2%)	2 (5%)	
Missing	1 (1%)	0 (0%)	1 (3%)		–	–	–	
Ethnicity n (%)*^				3.72				3.73
White British	75 (91%)	38 (87%)	37 (94%)		77 (93%)	39 (89%)	38 (97%)	
White Other	1 (1%)	1 (2%)	0 (0%)		1 (1%)	1 (2%)	0 (0%)	
Asian Pakistani	2 (2%)	1 (2%)	1 (3%)		2 (2%)	1 (2%)	1 (3%)	
Asian Other	3 (4%)	3 (7%)	0 (0%)		2 (2%)	2 (5%)	0 (0%)	
Mixed/Multiple ethnic groups	–	–	–		1 (1%)	1 (2%)	0 (0%)	
Missing	2 (2%)	1 (2%)	1 (3%)		–	–	–	
Employment status n (%)^				0.32				1.86
Retired	44 (53%)	23 (52%)	21 (55%)		45 (54%)	23 (52%)	22 (56%)	
Working full-time	20 (24%)	10 (23%)	10 (26%)		15 (18%)	7 (16%)	8 (21%)	
Working part-time	5 (6%)	3 (7%)	2 (5%)		10 (12%)	6 (14%)	4 (10%)	
Unable to work	12 (14%)	7 (16%)	5 (13%)		6 (7%)	3 (7%)	3 (8%)	
Do not work	–	–	–		6 (7%)	4 (9%)	2 (5%)	
Missing	2 (2%)	1 (2%)	1 (3%)		1 (1%)	1 (2%)	0 (0%)	
*Dialysis characteristics*								
Type of patient n (%)^				1.53				
Incident patient	54 (65%)	28 (64%)	26 (67%)		–	–	–	
Prevalent patient	6 (7%)	2 (4%)	4 (10%)		–	–	–	
Missing	23 (28%)	14 (32%)	9 (23%)		–	–	–	
Start of dialysis^				10.30				
Planned	52 (63%)	22 (50%)	30 (77%)		–	–	–	
Unplanned	4 (5%)	1 (2%)	3 (8%)		–	–	–	
Missing	27 (32%)	21 (48%)	6 (15%)		–	–	–	
Mode of dialysis n (%)^				1.76				
HD	50 (60%)	29 (66%)	21 (54%)		–	–	–	
PD	24 (29%)	10 (23%)	14 (36%)		–	–	–	
Missing	9 (11%)	5 (11%)	4 (10%)		–	–	–	
Type of access at pre-dialysis n (%)^				11.69				
AVF	27 (33%)	12 (27%)	15 (38%)		–	–	–	
Tesio line	7 (8%)	3 (7%)	4 (10%)		–	–	–	
PD catheter	21 (25%)	7 (16%)	14 (36%)		–	–	–	
Missing	28 (34%)	22 (50%)	6 (16%)		–	–	–	
*Clinical variables*								
**eGFR***M* (SD)	9.2 (3.3)	**10.0 (4.0)**	**8.5 (2.2)**	**0.04**	–	–	–	
Haemoglobin g/L *M* (SD)	107.9 (15.9)	109.9 (15.2)	105.8 (16.6)	0.27	–	–	–	
Serum albumin g/L *M* (SD)	37.9 (6.0)	39.0 (6.3)	36.7 (5.5)	0.10	–	–	–	
Comorbidity risk n (%)^				2.25				
Low	23 (28%)	12 (27%)	11 (28%)		–	–	–	
Medium	42 (50%)	20 (45%)	22 (56%)		–	–	–	
High	10 (12%)	6 (14%)	4 (10%)		–	–	–	
Missing	8 (10%)	6 (14%)	2 (5%)					
Primary renal diagnosis n (%)^				8.70				
Glomerulonephritis	10 (12%)	5 (11%)	2 (13%)		–	–	–	
Polycystic	9 (11%)	6 (14%)	3 (8%)		–	–	–	
Diabetes	7 (8%)	4 (9%)	3 (8%)		–	–	–	
Renal vascular disease	5 (6%)	4 (9%)	1 (3%)		–	–	–	
Hypertension	4 (5%)	2 (5%)	2 (5%)		–	–	–	
Pyelonephritis	3 (4%)	3 (7%)	0 (0%)		–	–	–	
Other	4 (5%)	3 (7%)	1 (3%)		–	–	–	
Uncertain	7 (8%)	4 (9%)	3 (8%)		–	–	–	
Missing	34 (41%)	13 (30%)	21 (54%)					
*Quality of life*								
WHOQOL General QOL	2.8 (0.9)	2.8 (0.9)	2.8 (0.8)	0.94	3.5 (0.9)	3.5 (0.9)	3.6 (0.9)	0.47
WHOQOL Physical	46.4 (21.9)	44.46 (23.1)	48.3 (20.7)	0.44	67.3 (21.3)	66.0 (21.1)	68.7 (21.6)	0.58
WHOQOL Psychological	61.7 (18.6)	60.1 (17.4)	63.2 (19.9)	0.48	66.0 (18.6)	64.5 (19.1)	67.5 (18.2)	0.50
WHOQOL Social	63.2 (21.1)	61.2 (20.6)	65.3 (21.7)	0.40	64.8 (16.7)	62.3 (18.3)	67.4 (14.6)	0.18
WHOQOL Environmental	67.4 (15.1)	65.6 (14.6)	69.2 (15.6)	0.29	67.8 (15.9)	65.0 (15.2)	70.8 (16.2)	0.11
*Psychological and relationship variables*								
HADS Anxiety	6.9 (4.3)	7.1 (4.3)	6.7 (4.3)	0.73	7.1 (4.0)	6.4 (3.8)	7.8 (4.2)	0.13
HADS Depression	6.8 (4.2)	6.9 (4.3)	6.7 (4.1)	0.83	5.1 (4.1)	5.4 (4.3)	4.7 (3.9)	0.46
Dialysis expectations	3.3 (0.7)	3.3 (0.7)	3.4 (0.7)	0.62	3.2 (0.5)	3.1 (0.4)	3.2 (0.6)	0.46
Accepting dialysis	3.3 (0.6)	3.3 (0.6)	3.3 (0.6)	0.82	3.4 (0.7)	3.4 (0.6)	3.4 (0.7)	0.71
Dyadic relationship characteristics	3.9 (0.6)	4.1 (0.6)	3.8 (0.7)	0.09	3.8 (0.6)	3.9 (0.6)	3.7 (0.5)	0.13
Relationship satisfaction	4.4 (0.9)	4.4 (0.9)	4.5 (0.9)	0.79	4.4 (0.7)	4.3 (0.8)	4.4 (0.7)	0.71
*Symptoms*								
Symptom severity	20.9 (11.5)	22.1 (13.3)	19.8 (9.5)	0.39	9.3 (8.7)	9.2 (9.1)	9.3 (8.4)	0.97
Number of symptoms	9.3 (4.2)	9.0 (4.8)	9.5 (3.6)	0.58	4.8 (3.6)	4.4 (3.8)	5.2 (3.4)	0.29

Note. *AVF* arteriovenous fistula, *HADS* Hospital Anxiety and Depression Scale, *HD* haemodialysis, *PD* peritoneal dialysis, *eGFR* estimated glomerular filtration rate, *QOL* quality of life, *WHOQOL* World Health Organization’s Quality of Life Short Form (BREF). Incident patient means a patient starting dialysis for the first time; prevalent refers to a patient who has been on a form of renal replacement therapy before but who intends to start dialysis due to a failing transplant. QOL was measured using the WHOQOL-BREF with scoring range of 1–5 for General QOL and 1–100 for its four domains. Anxiety and depression were assessed using the HADS, scoring range 0–21. Dialysis expectations, accepting dialysis, dyadic relationship characteristics and relationship satisfaction were assessed using study specific measures designed by the research team, each with a scoring range of 1–5. Symptoms were measured using the Palliative care Outcomes Scale – Symptoms (POS-S). Patients completed the renal version (17 items, severity symptom score range 0–68), and partners completed the general version (14 items, severity symptom score range 0–56). High scores on the WHOQOL-BREF indicate good QOL. High scores on dialysis expectations, accepting dialysis, DRC and relationship satisfaction suggest high expectations of dialysis, being accepting of dialysis, cohesive relationships characteristics between patients and partners, and satisfaction in the couple’s relationship. High scores on the HADS and POS-S suggest the presence of anxiety, depression and high symptom burden

* Ethnicity codes taken from those used in UK renal units

^ Chi-squared

Socio-demographic information on gender, age, relationship status, ethnicity, employment status, and highest educational level attained was self-reported at pre-dialysis. At the follow-ups, patients self-reported any changes in their dialysis treatment including mode of dialysis, access site (i.e., how they connected to their dialysis machine, e.g., catheter, fistula), renal unit, and if they had been hospitalized. Clinical data were collected from patients’ medical records by the SI in each renal unit at each time point. Clinical data included eGFR (baseline only), haemoglobin, serum albumin, dialysis adequacy (follow-ups only), comorbidities, primary renal diagnosis (PRD), and dialysis information (type of access: arteriovenous fistula [AVF], tesio line, PD catheter; mode of dialysis; start of dialysis: unplanned or planned; patient type: incident or prevalent; dialysis prescription; and dialysis status 3 months and 6 months after participating). Patients’ comorbidity data were scored following the guidelines of Davies et al. [26]. In this scoring method, active comorbidities are classified into one of seven domains (malignancy, ischaemic heart disease, peripheral vascular disease, left ventricular dysfunction, diabetes mellitus, systemic collagen vascular disease, and other significant pathology), and the patient’s comorbid risk level is attained by adding the number of domains affected (scoring range for risk 0–7: 0 = low, 1–2 = medium, ≥3 = high).

### Statistical analysis

Missing data were reviewed and descriptive statistics (means, standard deviations, percentages) were calculated for the QOL, socio-demographic and clinical variables. To determine if differences existed between participants who did and did not complete the study, t-tests were used for continuous variables and chi-squared for categorical variables.

Multilevel modelling (MLM) provides a robust yet flexible framework to estimate changes over time in nested structures, such as individuals within couples. Multilevel models estimated using maximum likelihood can accommodate missing data unlike ANOVA based methods which drop observations using listwise deletion [27]. In the context of repeated measures, this means that all available data for an individual will be used in estimating the model, rather than reducing the dataset to complete cases only.

For the present data structure, of repeated observations within individuals, nested within a social group (e.g., couple), a three-level mixed effects linear regression with random intercepts was used to estimate changes in WHOQOL general QOL. The MLM equation used for calculating the changes between groups (patients and partners) and over the follow-ups (F1 = pre-dialysis to 6 weeks, F2 = pre-dialysis to 12 weeks):

$$ \mathrm{QOL}={\upbeta}_0+{\upbeta}_1 Group+{\upbeta}_2 F1+{\upbeta}_3 F2+{\upbeta}_4 Group\ast F1+{\upbeta}_5 Group\ast F2+u+v+e $$

Then, a linear combination of parameters was used to examine whether partners’ QOL changed significantly from pre-dialysis to 6 weeks and pre-dialysis to 12 weeks. To aid in the interpretation of these interactions, the results of the models will also be displayed graphically using marginal mean plots to illustrate the models’ regression coefficients.

To analyse changes in the WHOQOL domains, the above equation and sequence was replicated for each domain by replacing the general QOL outcome variable with the transformed domain scores in each model.

To examine the association of baseline clinical and socio-demographic variables with changes in WHOQOL general QOL, separate two-level models for patients and partners with the interaction for time were conducted for each variable (Patients: haemoglobin, eGFR, serum albumin, mode of dialysis, type of patient, start of dialysis, access type in patients planning to start HD, PRD, comorbidity risk, gender, and age; Partners: gender and age):

$$ {\mathrm{QOL}}_{\mathrm{ij}}={\upbeta}_0+{\upbeta}_1{\mathrm{X}}_{\mathrm{j}}+{\upbeta}_2{F1}_{ij}+{\upbeta}_3{F2}_{ij}+{\upbeta}_4{\mathrm{X}}_{\mathrm{j}}\ast {F1}_{ij}+{\upbeta}_5{\mathrm{X}}_{\mathrm{j}}\ast {F2}_{ij}+{v}_j+{e}_{ij} $$

Paired sample t-tests were used to examine the differences in patients' scores over time within the facets of the WHOQOL physical domain at pre-dialysis and 12 weeks. A *p*-value of < 0.05 was considered significant. All statistical analyses were conducted using Stata (Version 15.1, Stata Corp, College Station, Texas, USA).

## Results

### Questionnaire characteristics

Missing data on the WHOQOL-BREF were minimal at each time point in the WHOQOL general QOL facet (pre-dialysis (T1): 2%, 6 weeks (T2): 2%, 12 weeks (T3): 0%) and across items of the four WHOQOL domains (T1: 11%, T2: 4%, T3: 4%). An exception was the item ‘How satisfied are you with your sex life?’ which had a higher percentage of missing data (T1: 11%, T2: 11%, T3: 15%). Questionnaires with > 20% missing data across the 26 items were not included in the analysis (T1: 6%, T2: 0%, T3: 0%). WHOQOL general QOL and its domain scores were only calculated if 80% of the items comprising it were completed.

### Participant characteristics

**Fig. 1:**
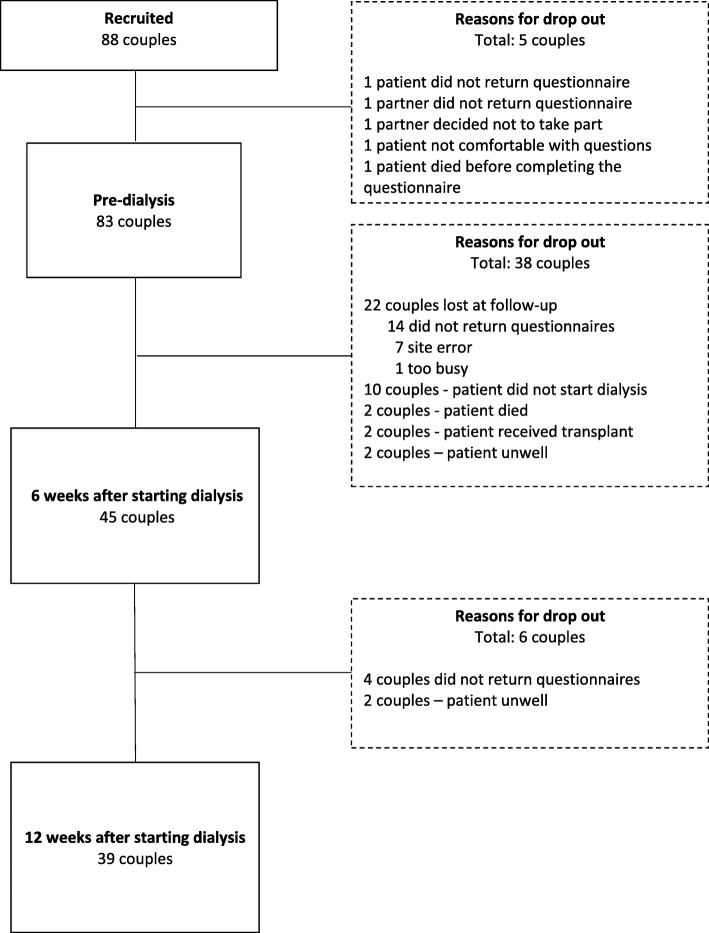
Flow chart showing recruitment and retention

Of the 153 patients invited to join the study, 91 (59%) consented. Reasons given for not participating were: the patient or partner did not feel well enough (*n* = 12), too busy to take part (*n* = 8), patient started dialysis before informed consent and the first questionnaire completed (*n* = 4), partner not interested in participating (*n* = 3), patient waiting to receive a transplant (*n* = 1), found the questions too intrusive (*n* = 1), and reason not offered (*n* = 25). Of the 88 couples who both consented, 83 couples returned their baseline questionnaires. Significant drop out (*n* = 38 couples, 46%) occurred from pre-dialysis to 6 weeks with 45 couples providing data at 6 weeks. Between the 6 and 12-week follow-up, a further 6 couples (14%) dropped out so that a total of 39 couples provided data across the study time points (see Fig. 1).

Comparisons between participants who completed the study and those who dropped out before follow-ups indicated minimal differences between these groups in terms of baseline socio-demographic, dialysis, clinical, QOL, psychological, relationship, or symptom variables. However, patients who only participated at baseline had a higher eGFR than those who completed the study (10.0 ± 4.0 vs 8.5 ± 2.2, *p* = 0.04). These baseline characteristics are provided in Table 1. Additionally, raw means and standard deviations of WHOQOL-BREF scores for patients and partners at baseline and the follow-ups are provided in Table 2.

**Table 2:** WHOQOL-BREF scores for patients and partners, raw mean (SD)

	*Patients*			*Partners*		
	Pre-dialysis	6 weeks	12 weeks	Pre-dialysis	6 weeks	12 weeks
	*n* = 83	*n* = 45	*n* = 39	*n* = 83	*n* = 45	*n* = 39
WHOQOL General QOL	2.8 (.9)	3.2 (.9)	3.3 (.9)	3.5 (.9)	3.3 (.8)	3.5 (.8)
WHOQOL Physical	46.4 (21.9)	49.8 (22.3)	54.9 (21.0)	67.3 (21.3)	66.8 (19.2)	67.9 (20.5)
WHOQOL Psychological	61.7 (18.6)	61.4 (21.0)	61.5 (18.8)	66.0 (18.6)	64.9 (15.8)	62.9 (20.1)
WHOQOL Social	63.2 (21.1)	63.8 (21.1)	66.6 (21.0)	64.8 (16.7)	65.0 (16.6)	65.5 (16.1)
WHOQOL Environmental	67.4 (15.1)	68.0 (19.9)	68.0 (16.4)	67.8 (15.9)	70.1 (16.6)	67.4 (17.2)

Note. *QOL* Quality of Life, *WHOQOL* World Health Organization Quality of Life BREF version. WHOQOL general is the mean of the Overall QOL and Satisfaction with health questions on the WHOQOL-BREF. Higher scores suggest better QOL

At 6 weeks after starting dialysis, 22 (49%) patients had begun conventional HD (4 h three times per week; standard UK guidelines), 13 (29%) were on PD, 1 (2%) used HD for 2 h twice per week, and for 9 (20%) patients their dialysis prescription was missing. Between pre-dialysis and the 6-week follow-up, 15 patients had moved to a new renal unit, 3 changed their type of access, 2 changed their dialysis mode, and 13 were hospitalized since starting dialysis. Between the 6 week and 12 weeks, 4 patients moved renal units, 2 changed their access types, 4 changed dialysis mode, and 4 were hospitalized. Mean dialysis adequacy was 1.45 ± 0.47 at 6 weeks and 1.38 ± 0.36 at 12 weeks; however, there was considerable missing data (T2: 32%, T3:69%).

The sample contained 166 participants at baseline, 90 at 6 weeks, and 78 at 12 weeks. This sample size enabled 80% power to detect a medium effect (F = 0.32) of changes in WHOQOL general and its domains, from pre-dialysis to each follow-up. Residuals were assessed and normally distributed in each of the random-effects models.

### Changes in WHOQOL general QOL

#### Patients

**Fig. 2:**
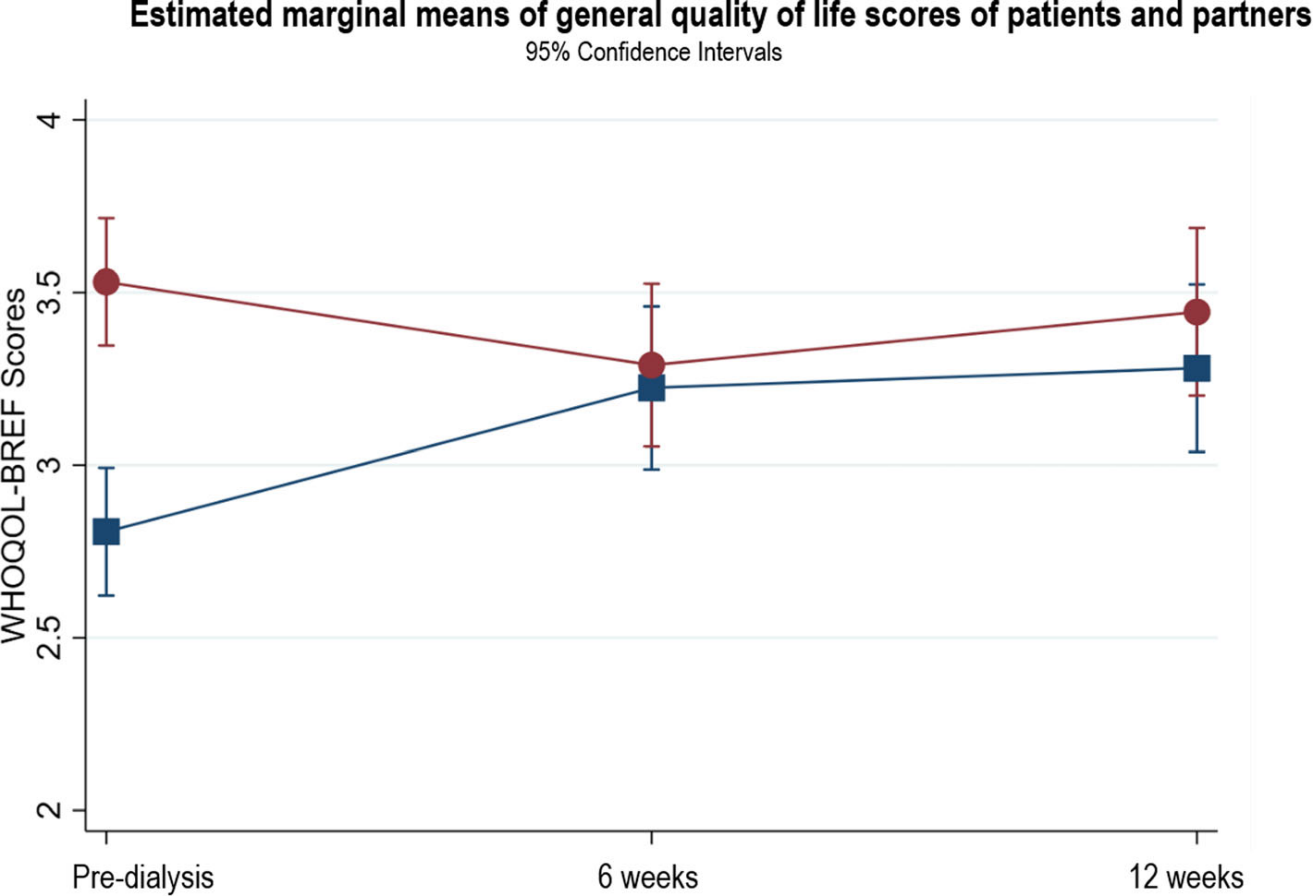
Estimated marginal means of general quality of life scores of patients and partners

Patients reported their general QOL as poor at pre-dialysis. At 6 weeks, patients’ QOL improved from poor to good (β = 0.42, *p* < 0.001, 95% CI 0.19, 0.65) with a slight further improvement at 12 weeks (β = 0.47, *p* < 0.001, 95% CI 0.24, 0.71). Figure 2 presents the marginal mean estimates of general QOL in patients and their partners predicted by the model. Additional file 1 provides the results of the three-level random intercept model for changes in general QOL for patients and partners.

#### Partners

Results from the linear combination indicated a statistically significant decline in partners’ general QOL scores from pre-dialysis to 6 weeks (β = − 0.24, *p* = 0.04, 95% CI -0.47, − 0.01). General QOL returned to pre-dialysis levels at 12 weeks after the patient started dialysis (β = − 0.09, *p* = 0.47, 95% CI 0.33, 0.15).

### Differences between WHOQOL general QOL in patients and partners

Patients reported significantly lower general QOL at pre-dialysis compared to their partners (β = 0.72, *p* < 0.001, 95% CI 0.51, 0.93). At 6 and 12 weeks, general QOL was not statistically different between patients or partners (Fig. 2).

### Changes in WHOQOL domains

#### Patients

**Fig. 3:**
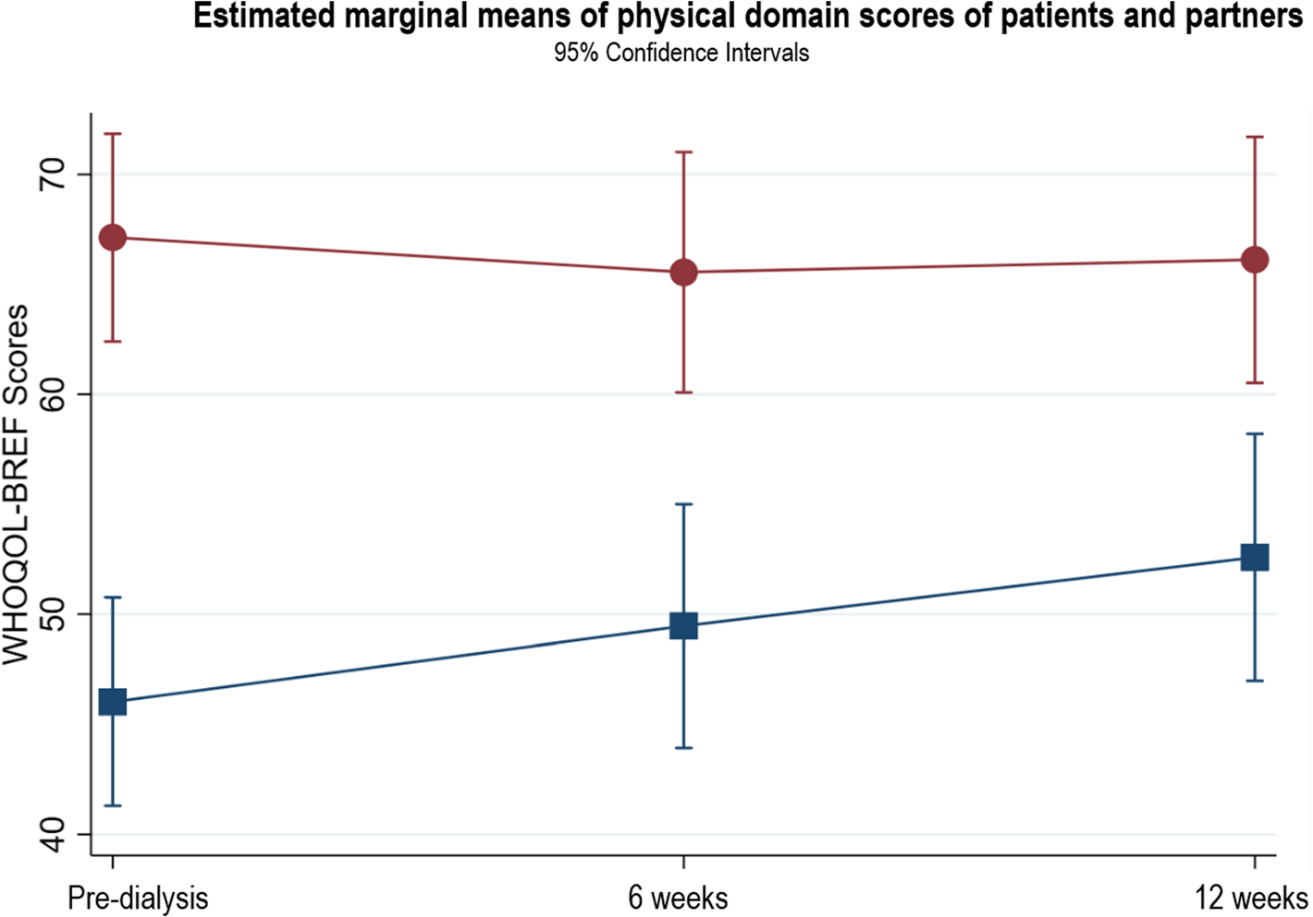
Estimated marginal means of physical domain scores of patients and partners

**Fig. 4:**
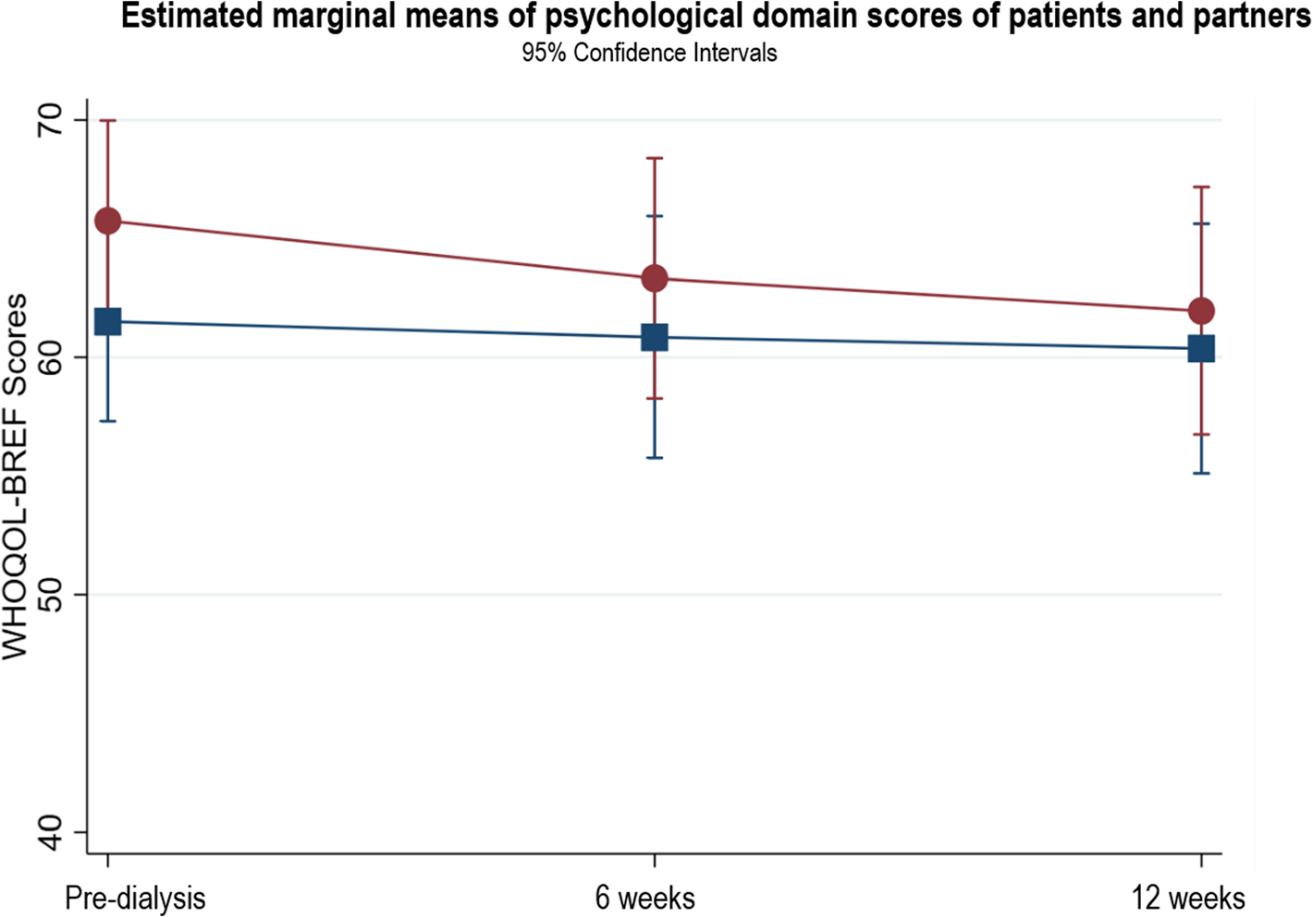
Estimated marginal means of psychological domain scores of patients and partners

**Fig. 5:**
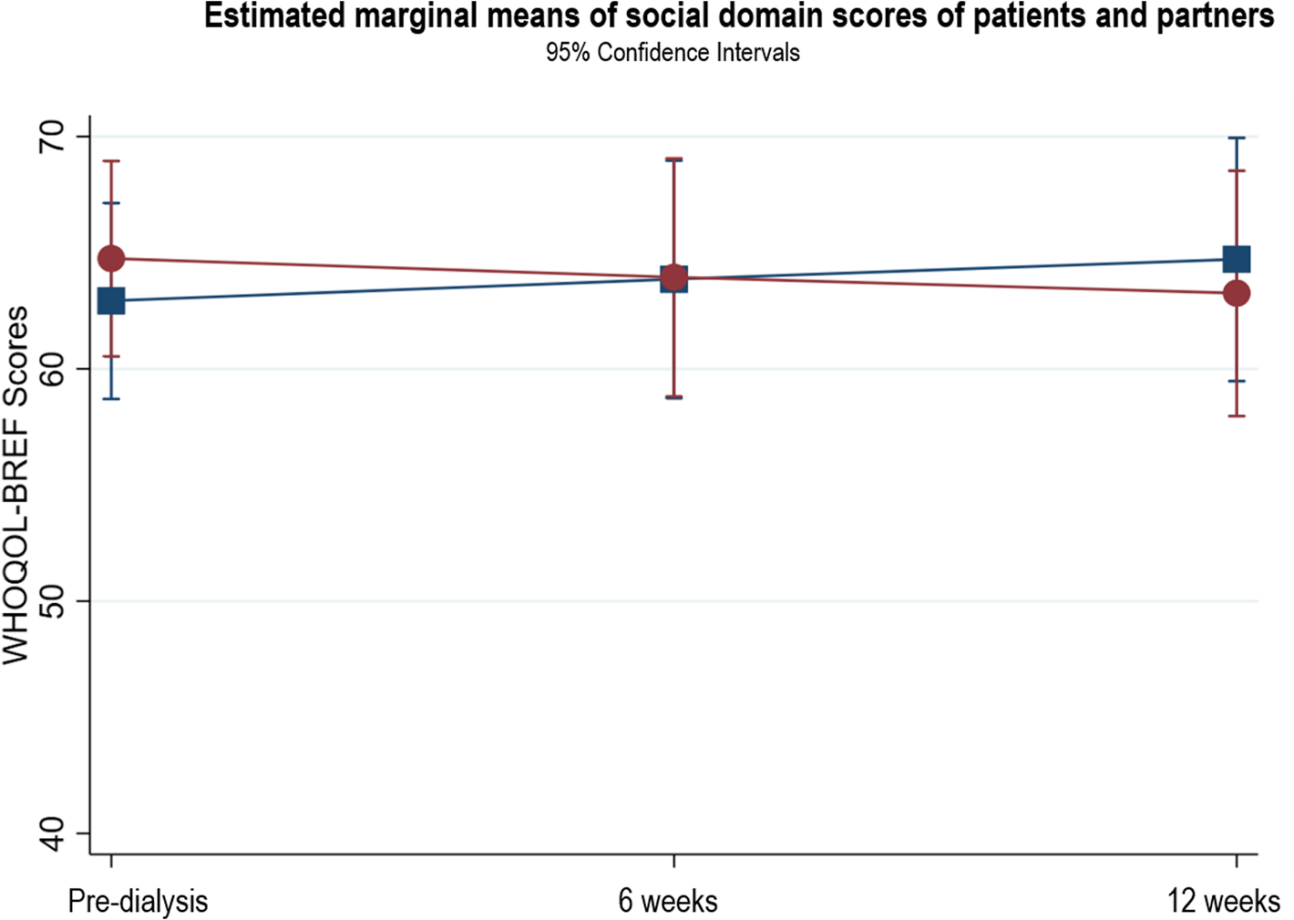
Estimated marginal means of social domain scores of patients and partners

**Fig. 6:**
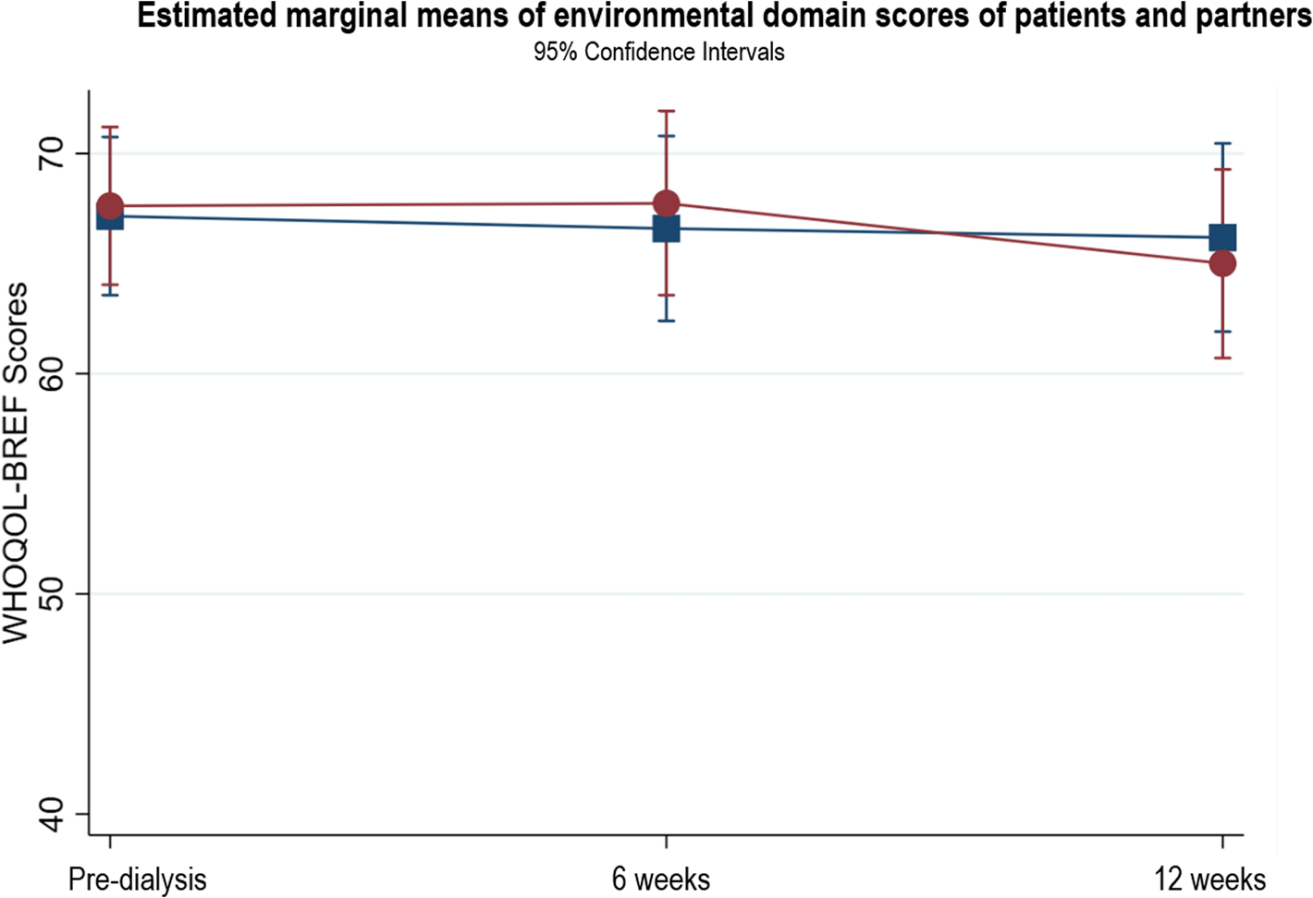
Estimated marginal means of environmental domain scores of patients and partners

The patients’ physical domain scores (Fig. 3) significantly improved between pre-dialysis and 12 weeks after starting dialysis (β = 6.56, *p* < 0.004, 95% CI 2.10, 11.03). Within the physical domain, there was a significant difference in sleep scores between pre-dialysis and 12 weeks (*n* = 39, T1: 2.78 ± 1.08; T3: 3.33 ± 1.11, *p* = 0.0028). This improvement indicates a change from poor sleep at pre-dialysis to good sleep at 12 weeks. No other domain scores showed statistically significant changes (see Figs. 4, 5, 6). In the psychological domain, scores decreased but still suggest good QOL. Social and environmental domain scores indicate good QOL, which remained stable over the study. Additional file 1 provides the results of the three-level random intercept models for changes in the domain scores for patients and partners.

#### Partners

No statistically significant changes occurred in any QOL domain (Figs. 3, 4, 5, 6). The physical, social and environment domain scores were stable and indicate good QOL. Similar to the patients, the partners’ psychological domain scores decreased from pre-dialysis to 12 weeks but reflect good QOL.

### Relationship between baseline clinical and socio-demographic variables and changes in WHOQOL general QOL

#### Patients

**Fig. 7:**
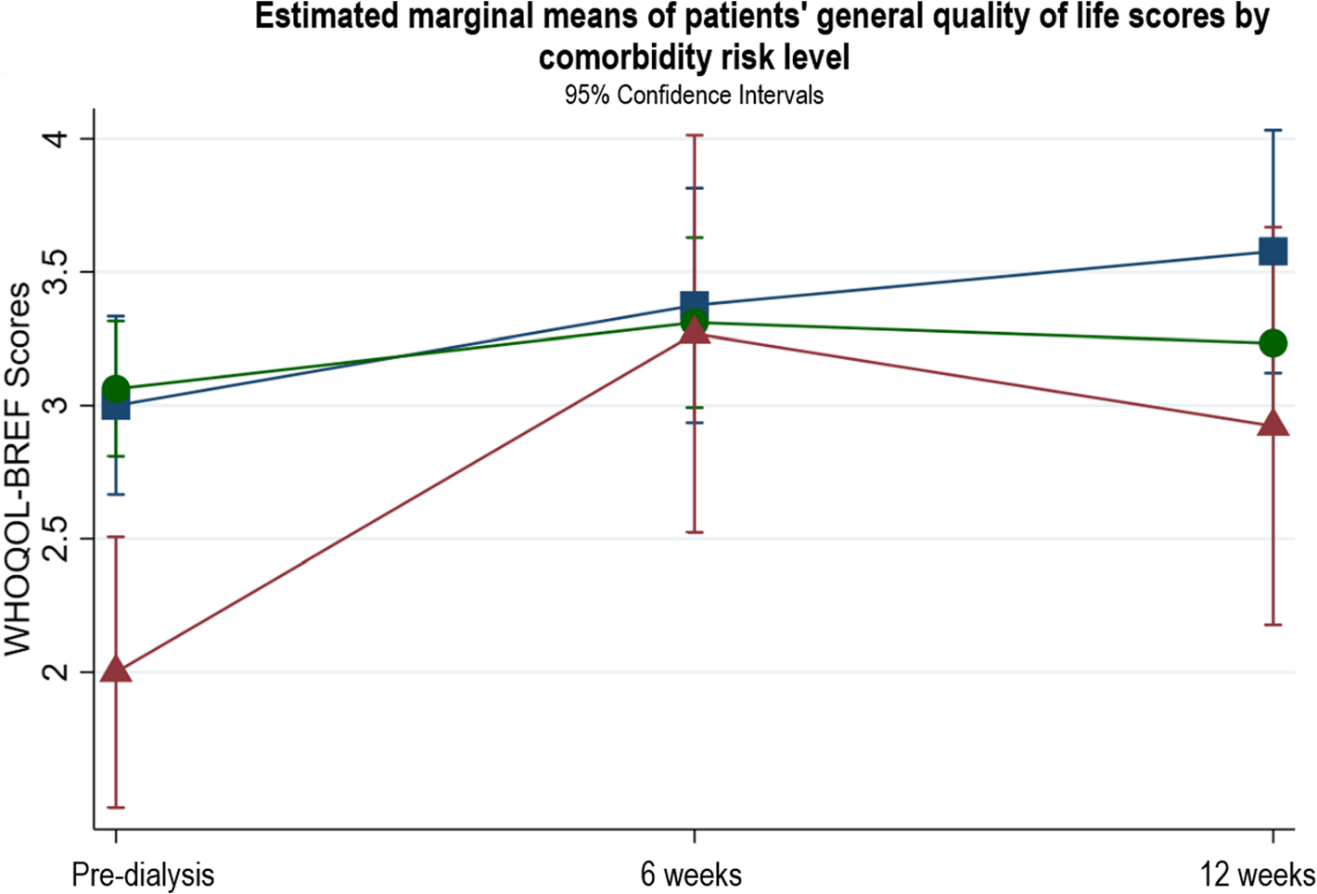
Estimated marginal means of patients’ general quality of life scores by comorbidity risk level

**Fig. 8:**
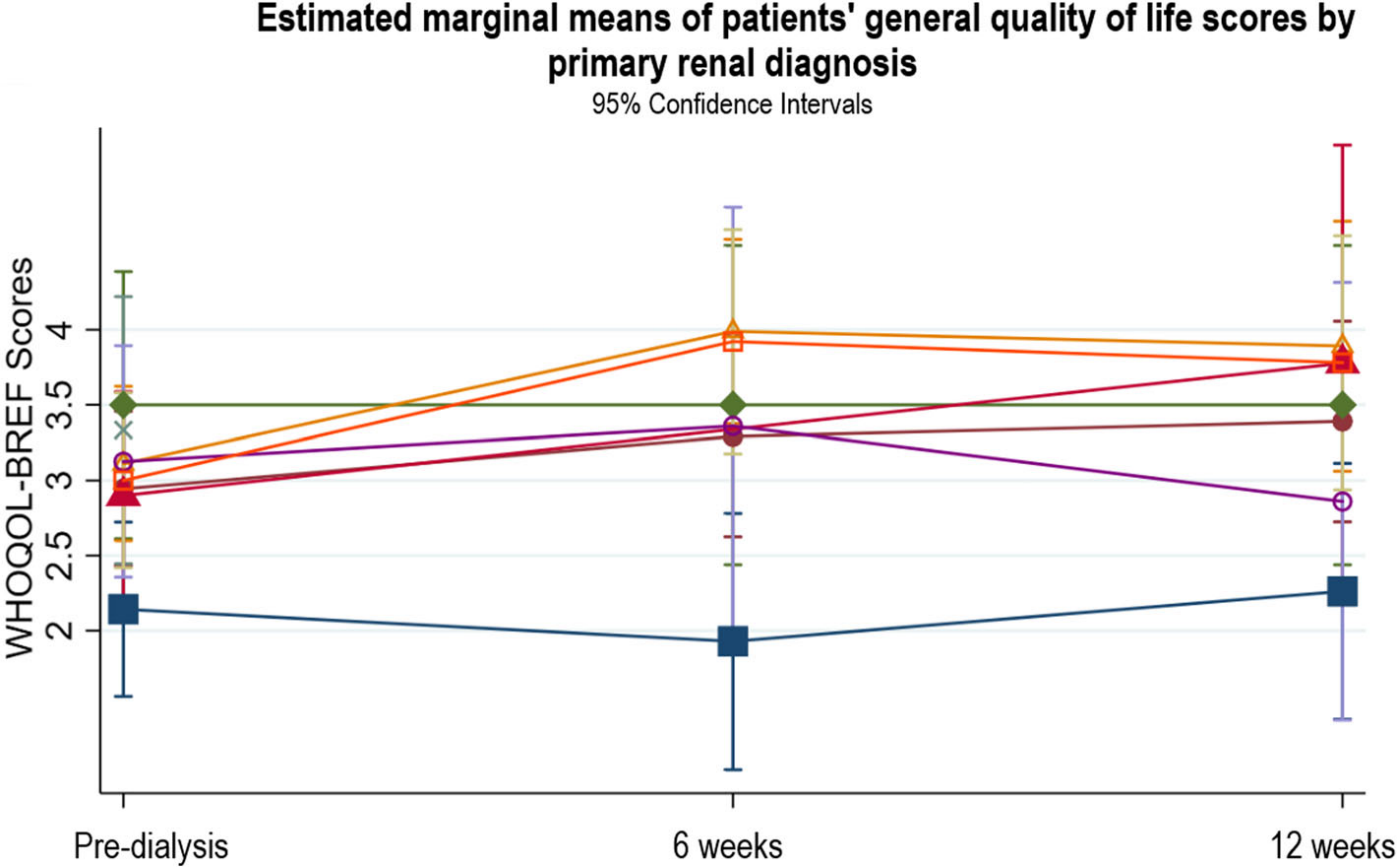
Estimated marginal means of patients’ general quality of life scores by primary renal diagnosis

**Fig. 9:**
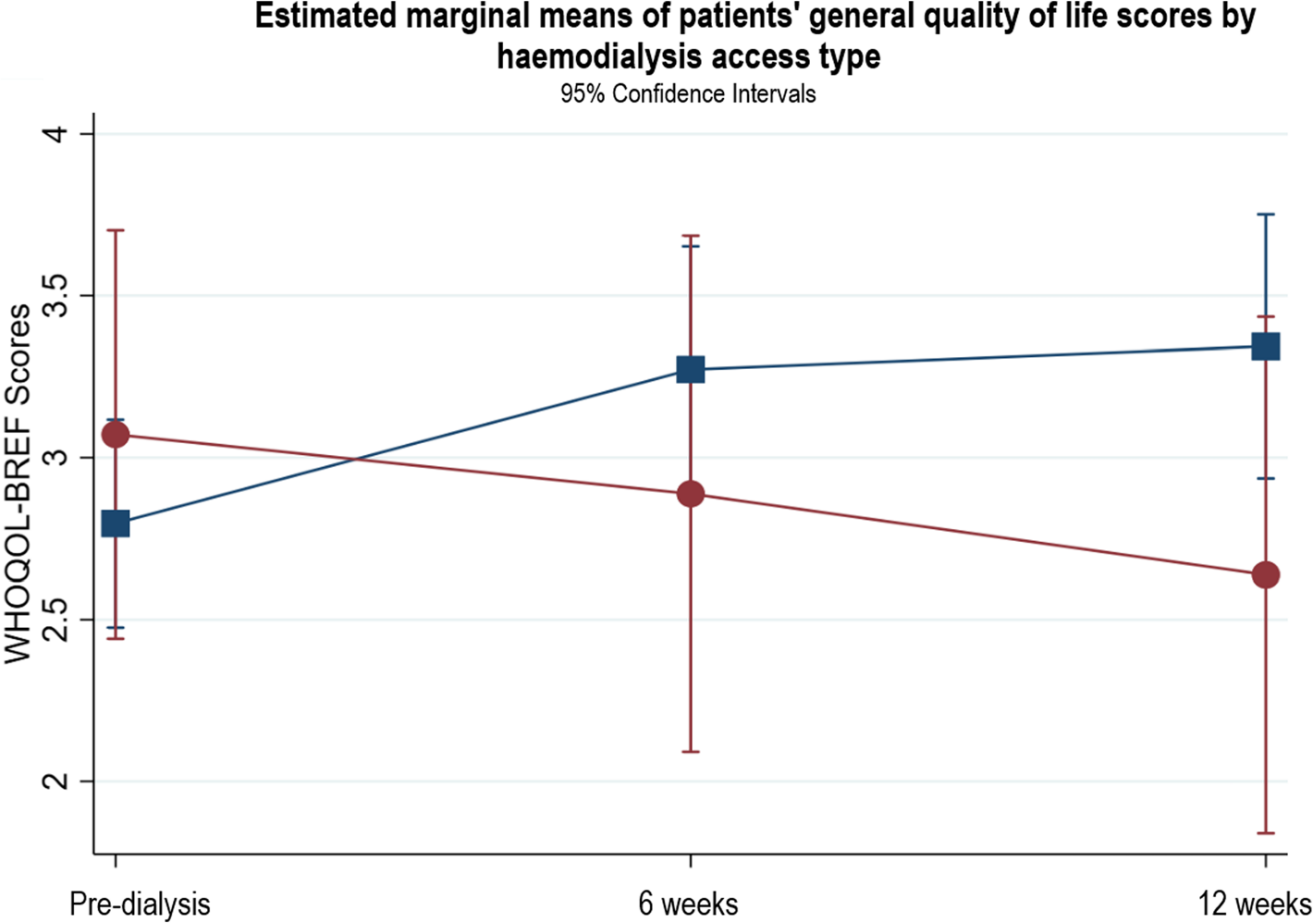
Estimated marginal means of patients’ general quality of life scores by haemodialysis access type

**Fig. 10:**
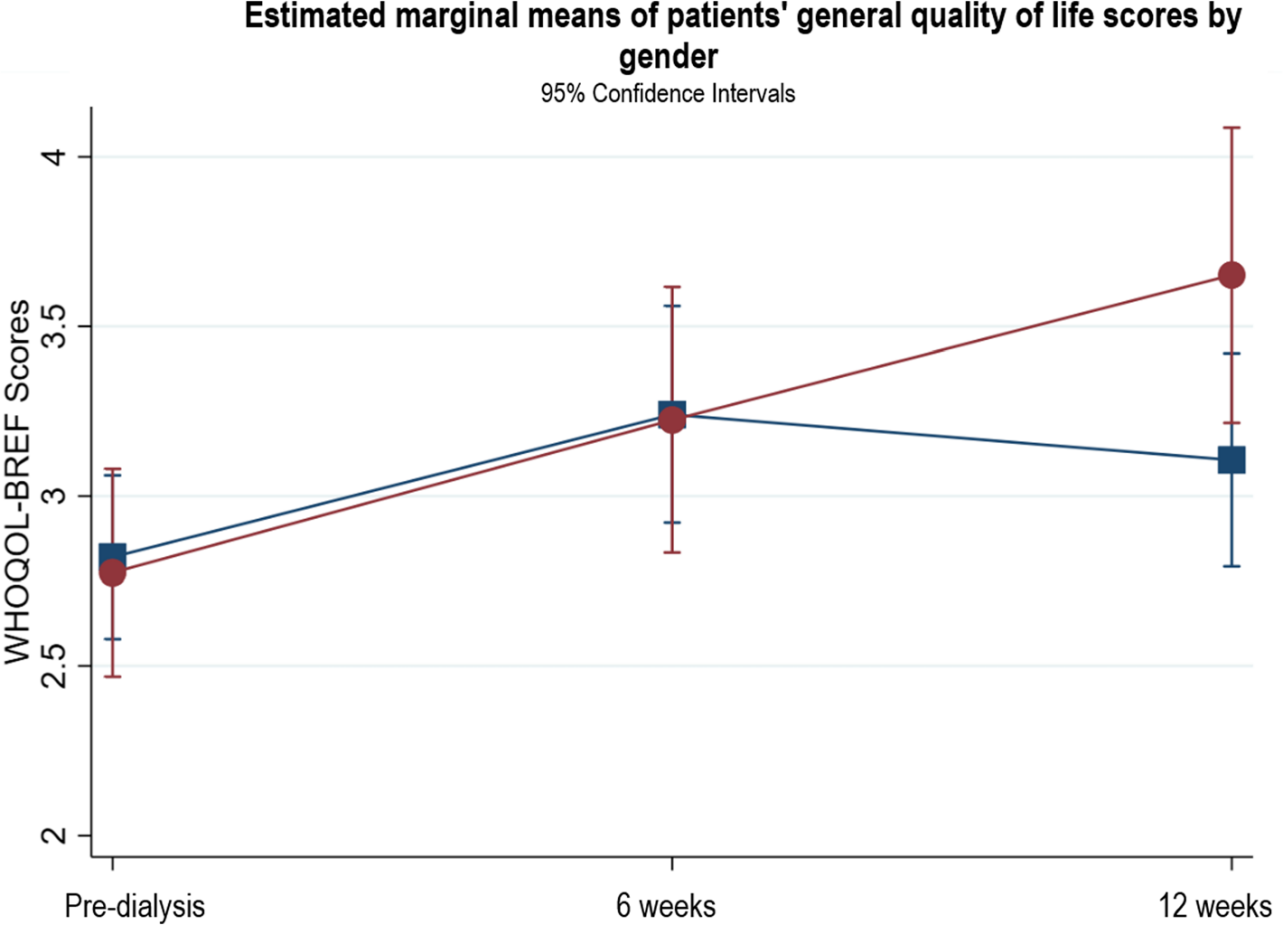
Estimated marginal means of patients’ general quality of life scores by gender

There were no significant associations between general QOL and baseline haemoglobin, eGFR, serum albumin, patient type, mode of dialysis, start of dialysis, or age. Comorbidity risk level, PRD, access type, and gender explained variations in the patterns of change in general QOL. For comorbidity risk level, there were no significant differences in changes in general QOL between patients with low and medium comorbidity risk. Patients with a high comorbidity risk had significantly worse general QOL at pre-dialysis compared to the low and medium risk groups (β = − 1.0, *p* = 0.001, CI -1.61, − 0.39), but their general QOL was not significantly different from the low or medium risk groups at 6 and 12 weeks (Fig. 7). Patients with a PRD of diabetes had poorer QOL at pre-dialysis which did not improve after starting dialysis which differed to those with other PRDs (see Fig. 8). In terms of access type in patients planning to start HD, there were no significant differences in QOL between patients with AVF or tesio line at pre-dialysis or 6 weeks (see Fig. 9); however, from pre-dialysis to 12 weeks, general QOL worsened in patients with a tesio line compared to those with AVF (β = − 0.98, *p* = 0.04, CI -1.90, − 0.06). In terms of gender, there were no significant differences in general QOL between males and females at pre-dialysis and 6 weeks (see Fig. 10); however, from pre-dialysis to 12 weeks female patients reported significant improvements in general QOL compared to male patients (β = 0.59, *p* = 0.03, CI 0.48, 1.13). Additional file 2 shows the results of the two-level random intercept models for changes in WHOQOL general QOL on the basis of baseline clinical and socio-demographic variables in patients and partners.

#### Partners

There were no significant associations with changes in general QOL on the basis of age or gender.

## Discussion

This study is the first to describe and examine QOL in patients and their partners as patients transition onto dialysis. Patients’ general QOL improved over the transition from pre-dialysis to dialysis, which remained stable at 12 weeks after starting dialysis. This change marks an important perceptual shift from poor to good QOL. Even patients with a high comorbidity risk experienced significant improvements in general QOL during this period. However, patients with a PRD of diabetes did not report improvements in general QOL. Furthermore, our data show that female patients and patients with AVF reported significant improvements in their general QOL compared to male patients or those with tesio lines. In particular, patients’ physical QOL also improved from pre-dialysis to 12 weeks, moving from poor to moderate physical QOL. Within the physical domain, better sleep quality was the key improvement. In contrast, partners reported impaired general QOL after patients had just started dialysis. However, it had improved by 12 weeks and returned to a level similar to that reported at the pre-dialysis stage. Partners’ general QOL remained good throughout the transition period, despite worsening at 6 weeks. In the psychological, social and environmental QOL domains, patients and partners reported good QOL, and this remained stable throughout the transition period.

These findings extend our understanding of changes in QOL in both members of a couple at the time that a patient starts dialysis. Importantly, it provides new information about what happens to QOL in the critical early weeks after initiating dialysis. Comparisons with existing research are difficult to interpret as the only other longitudinal study [21] involving patients and their partners over the transition onto dialysis administered the SF-36, which is a measure of health status and functioning rather than a QOL measure. Consequently, direct comparisons between the SF-36 and the WHOQOL-BREF cannot be made. At pre-dialysis, patients in the present study only had poor QOL in the general facet and the physical domain, whereas the patients on PD studied in Fan et al. [21] reported their physical and mental functioning as poor at this stage. Furthermore, partners in the present study reported WHOQOL-BREF general QOL and all domains as good at pre-dialysis, whereas the partners in Fan et al. [21] reported impaired physical and mental functioning.

As to the impact of starting dialysis on patients and their partners, our findings also contrast those reported by Fan et al. [21]. In the present study patients and their partners reported changes in their general QOL and patients’ physical QOL, but in Fan et al. [21] only the social functioning component of mental health improved after one year. It is possible that our study might also have detected a change in the social domain if follow-ups had been conducted 1 year after starting dialysis. While interpretations across the studies are limited, the present study’s findings fill an important gap in our knowledge about the impact of starting dialysis on the QOL of patients and their partners during the critical first 12 weeks.

Our findings contrast with studies in the wider ESRD literature, which report patients and partners’ physical and mental functioning as impaired, as measured by the SF-36 or SF-12 [28–36]. Our findings differ from the literature being one of the few studies conducted in the UK where patients have access to structured pre-dialysis pathways (i.e., specialist clinics which provide information on renal replacement therapy, diet, symptom management, and lifestyle advice about ESRD to patients and their families; 50% of patients start dialysis with access in place [37]). A majority of patients in the present study started dialysis in a planned manner and had access to structured pathways and pre-dialysis education, which are linked with positive patient outcomes [38]. Our findings highlight the need for on-going support to partners during the transition onto dialysis to prevent it impairing their QOL at this time.

To our knowledge, only two studies exist which have used the WHOQOL-BREF in patients with ESRD and their partners [39, 40]. Our findings indicate that patients and partners reported good QOL in all domains of the WHOQOL-BREF at 12 weeks contrasting with studies by Al-Rawashdeh et al. [40] and Anees et al. [39]. Al-Rawashdeh et al. conducted a cross-sectional study with 123 Jordanian patients on HD and their partners. Only partners reported good QOL in every domain, whereas patients only reported good social QOL. A cross-sectional study by Anees et al. [39] in Pakistan assessed 125 patients on HD and 50 of their family members. The partners studied by Anees et al. reported better QOL in three domains (physical, psychological and social) than patients whose QOL was poor or moderate in each of these domains. Comparing patients in the current study with Anees et al.*,* we conclude that our patients had better QOL across the domains. Partners in both studies had similar QOL in the same three domains. However, partners in the present study rated their environmental QOL better than partners in Anees et al. These comparisons should be made cautiously as our UK sample differs in its cultural context, ESRD practices, and renal  service features.

A growing number of studies have used the WHOQOL-BREF in patient-only samples. These too were conducted in cultural and renal contexts that may differ significantly from the UK. One study, conducted in Korea, used the WHOQOL-BREF with patients in pre-dialysis care for ESRD [41]. Comparing the pre-dialysis scores of patients in the two studies, our patients reported better QOL in the psychological, social and environment domains, but poorer physical QOL. Other studies in the field were conducted with patients established on dialysis [42–45]. Comparisons with their findings and ours indicate that QOL is similar, although patients in the present study reported slightly higher QOL. Similarity between physical QOL in this study and these others with patients established on dialysis indicates that physical QOL does not continue to improve as time on dialysis increases. Dialysis only partly removes the toxins from the body [8]; therefore, improvements in physical QOL due to dialysis may only be noticeable to patients initially.

Only one study by Kang et al. [46], conducted in Singapore, utilised the WHOQOL-BREF to assess QOL at baseline and 1 year in 44 partners of patients established on PD. Compared to Kang et al.*,* partners in the current study had similar general QOL, psychological and social QOL scores but much poorer physical QOL. The average age of partners in the current study was 63 years (±15 years), whereas in Kang et al. the average was 38 years (±6 years), which may explain the difference in physical QOL. Interestingly, partners in Kang et al. reported significant impairments in psychological QOL during the study. In the current study, partners’ psychological QOL had a downward trend which may indicate that dialysis and its related stressors have a delayed negative impact on their QOL. In comparison to other UK research and in the wider caregiving literature, physical QOL of the partners in this study are comparable to those found in healthy participants, whereas psychological, social and environmental domains of QOL are more similar to carers of elderly patients [14].

In the present study, we found changes in patients’ general QOL were moderated by comorbidity risk level, PRD, access type in patients planning to start HD, and gender. Our findings that poorer QOL was associated with patients with diabetes and having a tesio line echo that of the wider literature [8, 47, 48]. However, patients with a high comorbidity risk reported significant improvements to their general QOL that matched the low and medium risk groups, and female patients reported their QOL was better than male patients. These findings differ from that in the ESRD literature where high comorbidity and female gender are commonly related to poor QOL [47, 49]. However, the relationship between the moderator variables and general QOL should be interpreted cautiously due to the small sample size and missing data in four clinical variables (type of patients, start of dialysis, access type, and PRD) which means very little information could be used to compute the 6 and 12 week estimates.

Improvements in the patients’ physical QOL scores was due to better quality sleep. Other studies have found improvements in sleep quality, assessed the using 3 items forming the sleep domain in the CHEQ instrument; however, only a minority of patients (< 20%) in these other studies reported better sleep [36, 50]. That the patients in the current study reported improvements in their sleep at 12 weeks provides evidence as to when patients may experience benefits from dialysis.

In this study we selected the WHOQOL-BREF because it includes a facet on general QOL and items within the four domains address topics important to patients with ESRD and their partners. Given the active debate about how to measure QOL in the ESRD field [12], we analysed the two items comprising the WHOQOL general QOL facet as our primary outcome variable. Unlike the SF-12 and SF-36, they ask participants to rate their overall QOL *and* satisfaction with their health. These two global items from the WHOQOL general QOL facet may be a useful indicator for studying QOL at specific time intervals and between groups [51]. Furthermore, the four domains of QOL in the WHOQOL-BREF complement the life areas described by Jablonski [5] as being affected in the crisis phase. The WHOQOL-BREF also includes a question on sex-life, which some participants in the study did not answer. This finding suggests people may be hesitant to discuss their sex-life, even though it may be important to their QOL and impacted by ESRD. This study’s findings offer a new perspective on QOL in patients and their partners during early dialysis and suggests that there are significant changes in QOL which warrant further investigation.

A limitation of this study is the small final sample size. The recruitment period of 1 year yielded a sample of 83 couples. However, due to significant drop-out between pre-dialysis assessment and subsequent follow-ups, only 39 couples provided data at all three time points. Despite significant attrition, the only significant difference between the baseline characteristics of those who dropped out and those who completed the study was patients’ eGFR. All the patients were deemed to start dialysis within 2 months, hence the similarity between their overall symptoms, clinical and biochemical status. There is little to suggest that patients who completed the study were any different to those who did not; however, it is possible that the higher eGFR in those who dropped out (10.0 vs 8.5) indicates that they felt better in general and therefore may have been less likely to engage with ESRD research about starting dialysis. Future studies could benefit from collecting additional indices on blood, urea, and nitrogen which may provide a fuller picture of differences between patients and changes after starting dialysis.

Although there was considerable drop-out which effects overall power, using a random effects MLM allowed us to utilise all available data at the three time points which increased the precision of our estimates [27]. In addition, 93% of the participants were White British. This homogeneity limits the generalizability of findings to an ethnically diverse UK population. Follow-ups at 6 and 12 weeks may have been too soon after starting dialysis to detect significant changes in all QOL domains. However, these first 12 weeks are under-researched. The decision about timing was consolidated following consultation with a renal research patient-public involvement group, and participants from a previous qualitative study.

In this study only patients who were in a spousal-type relationship and whose partner also consented to take part were eligible. The wider chronic illness literature has suggested that recruiting and retaining couples in research requires significant additional time, resources, and multiple contacts [52]. These issues should be taken into consideration when designing future couple-based research in ESRD. Furthermore, when screening potential patients, we experienced issues identifying those with a partner as this level of detail is often not routinely recorded. Although we expected approximately 60% of patients to have partners, many sites found it to be as low as 30%. In most renal units, this type of information is not easily accessible, nor is it monitored at the national level. Without accessible data about partners, who are often primary informal caregivers to patients with ESRD, it is difficult to map an accurate picture of the caregiving landscape in the ESRD population. Therefore, we recommend that a mapping exercise is conducted across UK renal units to more comprehensively describe this area. Furthermore, we suggest that information on partners is routinely monitored and reported in a standardized way.

The major strength of our study is that it focuses on the changes in QOL during the crisis phase of dialysis. By zooming in on this time frame, we have exposed the complex effects of starting dialysis, improving patients’ QOL but initially impairing the QOL of their partners. At the same time, we demonstrate the need to include partners in research if we are to better appreciate the broader impact of treatments. This research provides a foundation to begin  better understanding QOL in patients and their partners; however, future research with more couples conducted over a longer time frame may uncover additional changes that occur as they adjust to the impact of treatment. Future research which examines factors which predict changes in QOL could also offer valuable insight.

## Conclusion

Our findings offer encouraging signs that patients’ general and physical QOL improve after starting dialysis. However, it also indicated that patients have impaired QOL before starting dialysis and that their partners’ general QOL diminishes in the initial weeks after the patient starts dialysis. It is promising that starting dialysis did not negatively impact the domains of QOL in patients or their partners.

## Supplementary information

**Additional file 1.** Results of the multilevel models of changes in QOL in patients and partners and results from the linear comparison of parameters analysis in partners.

**Additional file 2.** Results of the multilevel models of changes in QOL scores of patients and partners in relation to baseline clinical and socio-demographic variables.

## Data Availability

The dataset supporting the conclusions of this article are available from the corresponding author on reasonable request.

## Abbreviations

AVF: Arteriovenous fistula
CI: Confidence interval
eGFR: Estimated glomerular filtration rate
ESRD: End stage renal disease
F1: Pre-dialysis to 6 weeks after starting dialysis
F2: Pre-dialysis to 12 weeks after starting dialysis
HD: Haemodialysis
MLM: Multilevel modelling
NHS: National Health Service
NIHR: National Institute for Health Research
PD: Peritoneal dialysis
QOL: Quality of life
SF-12: Short Form Health Survey-12 items
SF-36: Short Form Health Survey-36 items
SI: Site investigator
T1: Pre-dialysis
T2: 6 weeks
T3: 12 weeks
WHOQOL-BREF: World Health Organization QOL 26-item questionnaire
