# Correction to: Quality of life improved for patients after starting dialysis but is impaired, initially, for their partners: a multi-centre, longitudinal study

**DOI:** 10.1186/s12882-020-01858-x

**Published:** 2020-07-06

**Authors:** Currie Moore, Lesley-Anne Carter, Sandip Mitra, Suzanne Skevington, Alison Wearden

**Affiliations:** 1 School of Health Sciences, Division of Psychology and Mental Health, Manchester Centre for Health Psychology, University of Manchester, Manchester, UK; 2 Manchester Academic Health Science Centre, University of Manchester, Manchester, UK; 3 Division of Population Health, Health Services Research & Primary Care, University of Manchester, Manchester, UK; 4 Manchester University NHS Foundation Trust, Manchester, UK; 5 NIHR Devices for Dignity MedTech Cooperative, Sheffield, UK

**Correction to: BMC Nephrology (2020) 21:185**

**https://doi.org/10.1186/s12882-020-01819-4**

Following publication of the original article [1], it was noted that due to a typesetting error the figure legends for Figs. 2, 3, 4, 5, 6, 7, 8, 9 and 10 were missing.

**Fig. 2:**
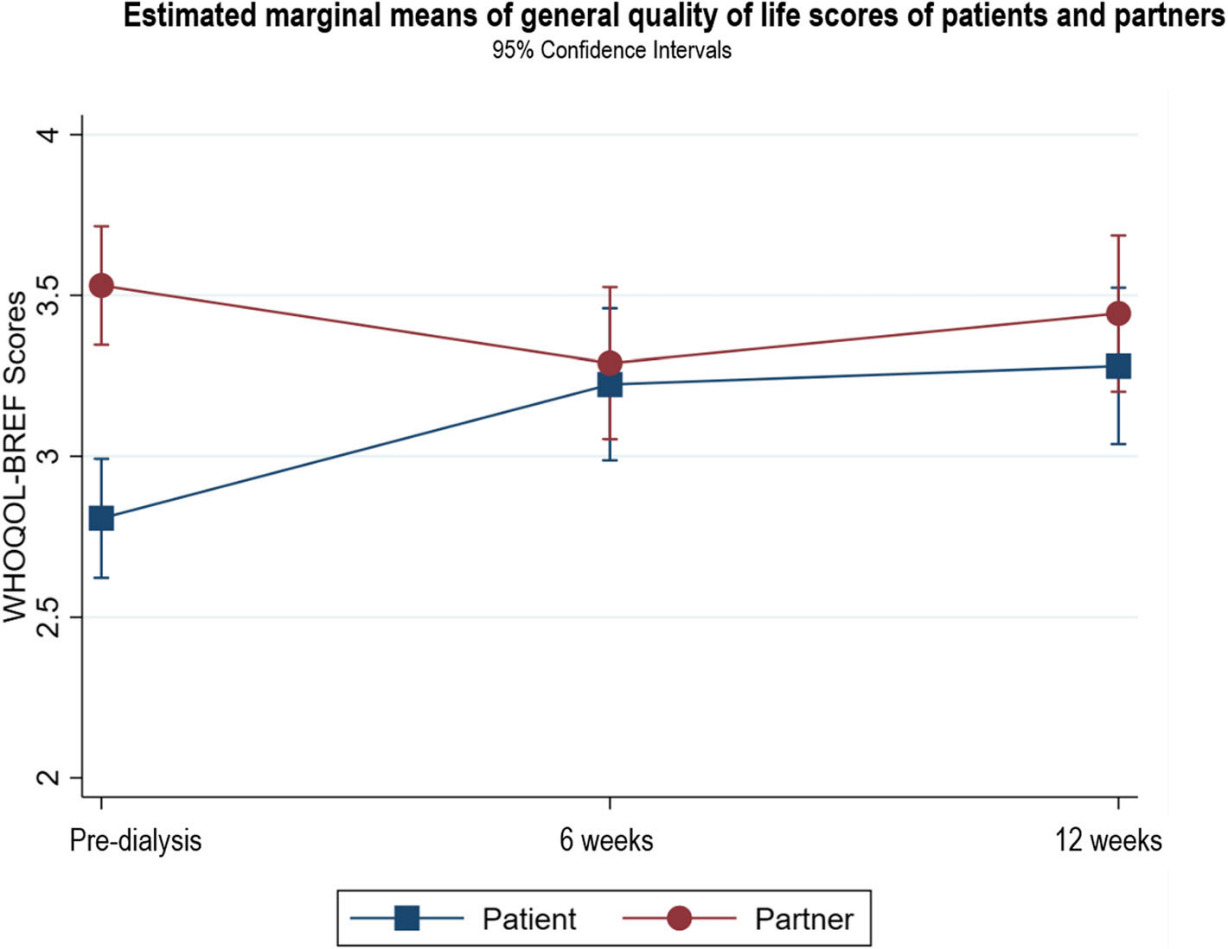
Estimated marginal means of general quality of life scores of patients and partners

**Fig. 3:**
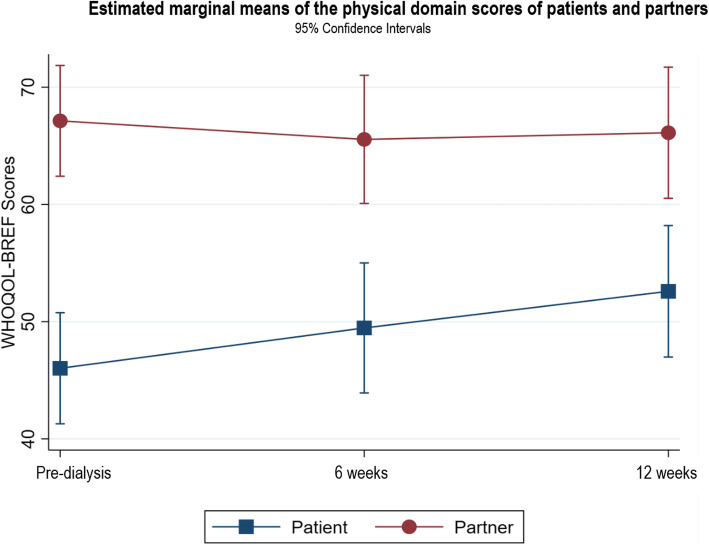
Estimated marginal means of physical domain scores of patients and partners

**Fig. 4:**
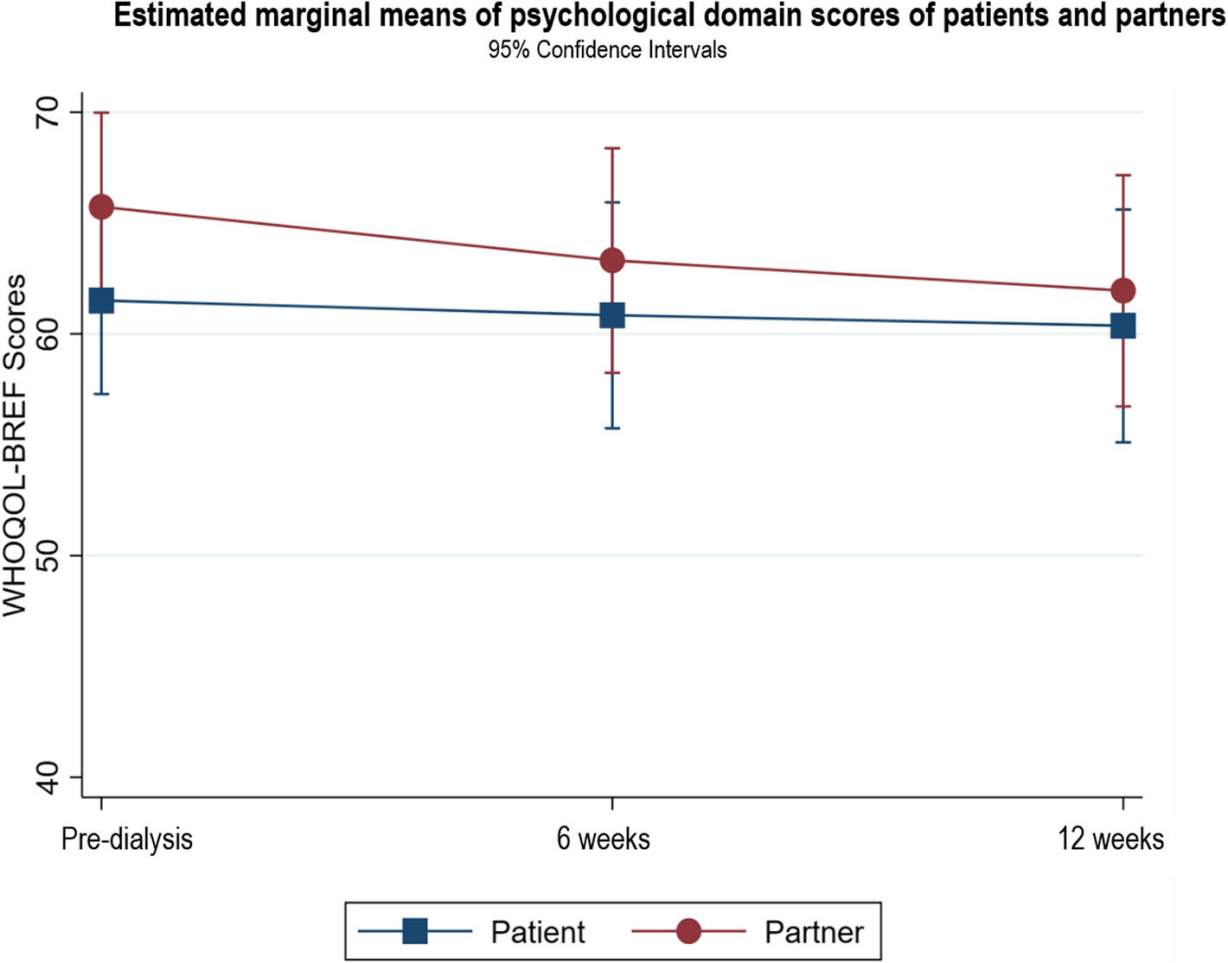
Estimated marginal means of psychological domain scores of patients and partners

**Fig. 5:**
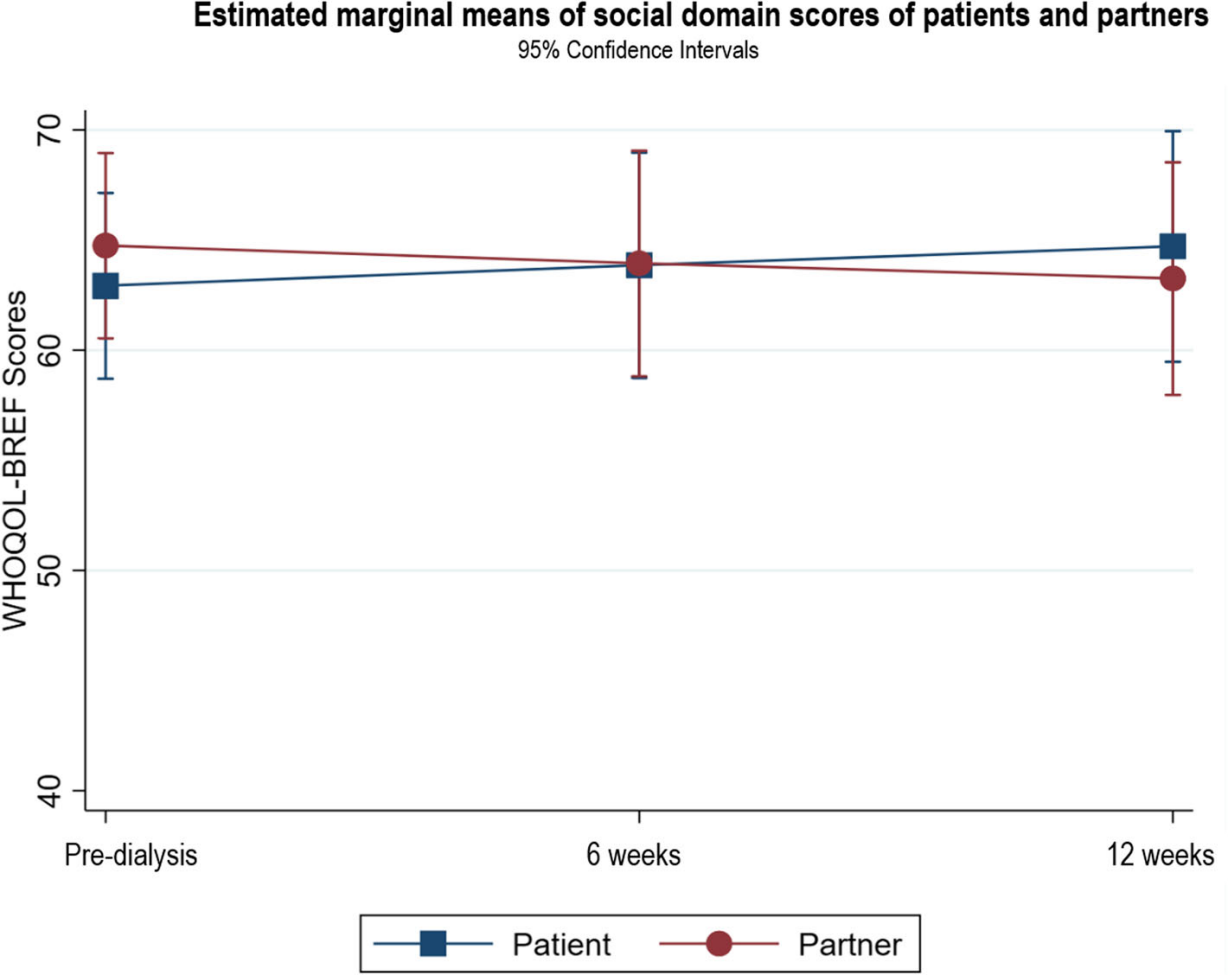
Estimated marginal means of social domain scores of patients and partners

**Fig. 6:**
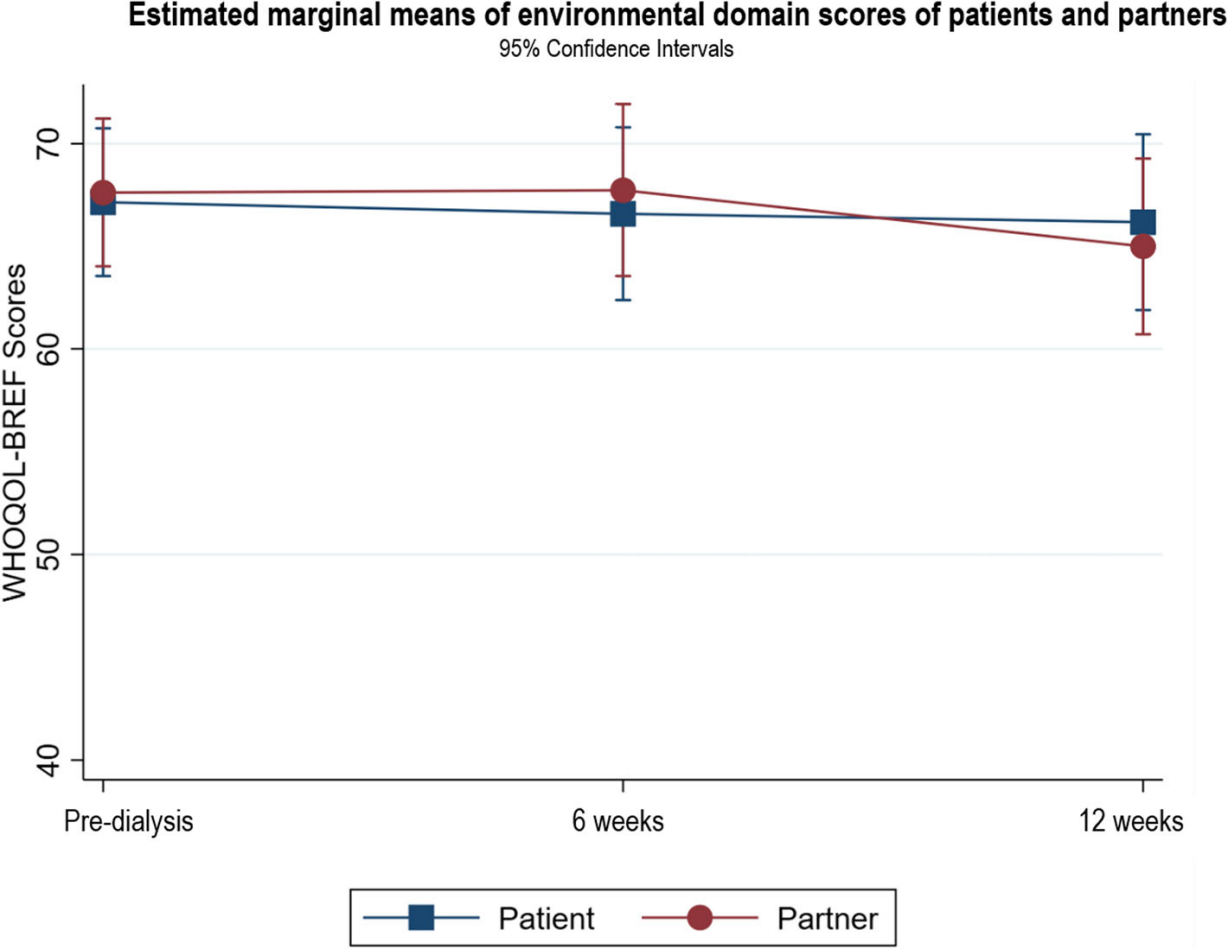
Estimated marginal means of environmental domain scores of patients and partners

**Fig. 7:**
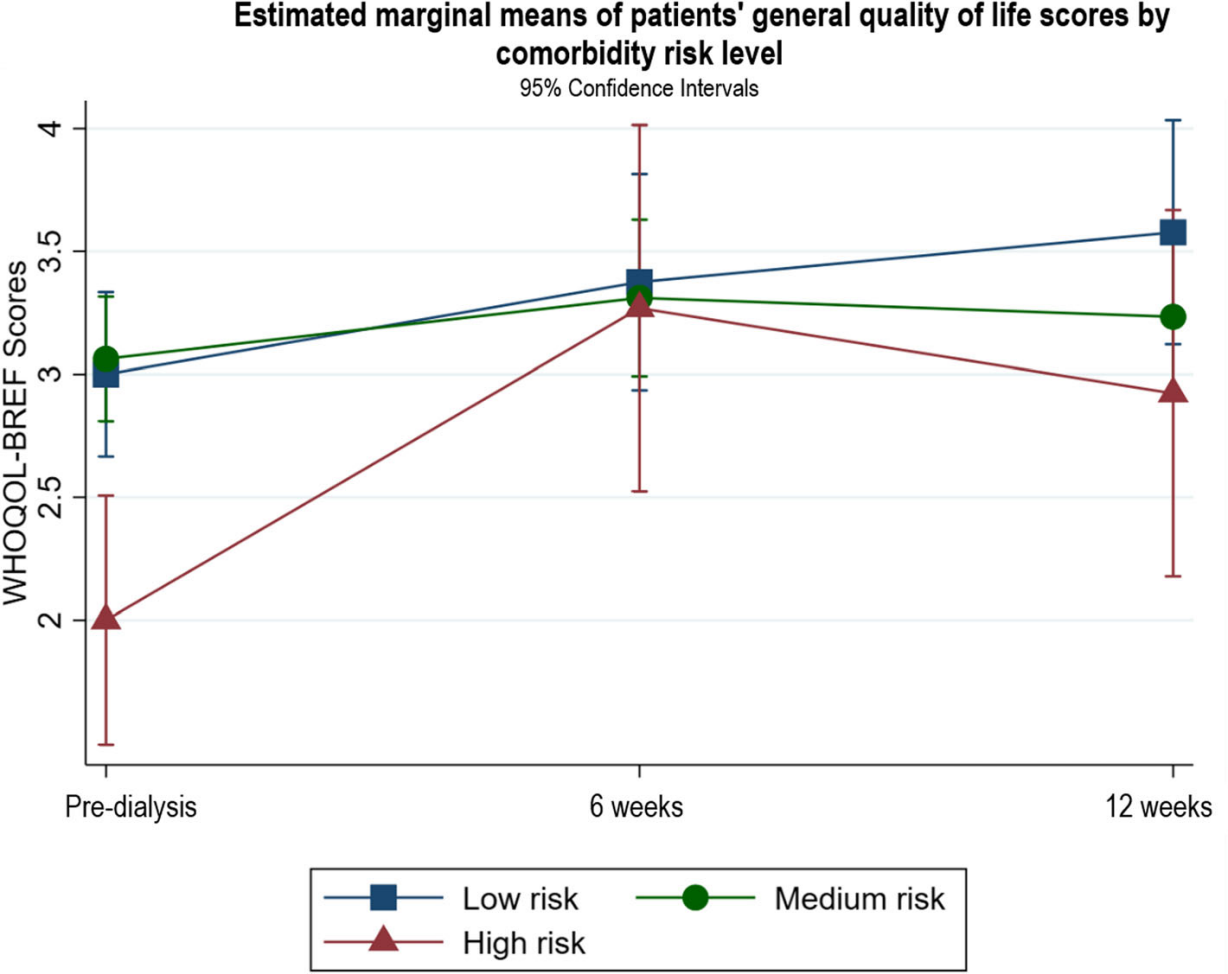
Estimated marginal means of patients’ general quality of life scores by comorbidity risk level

**Fig. 8:**
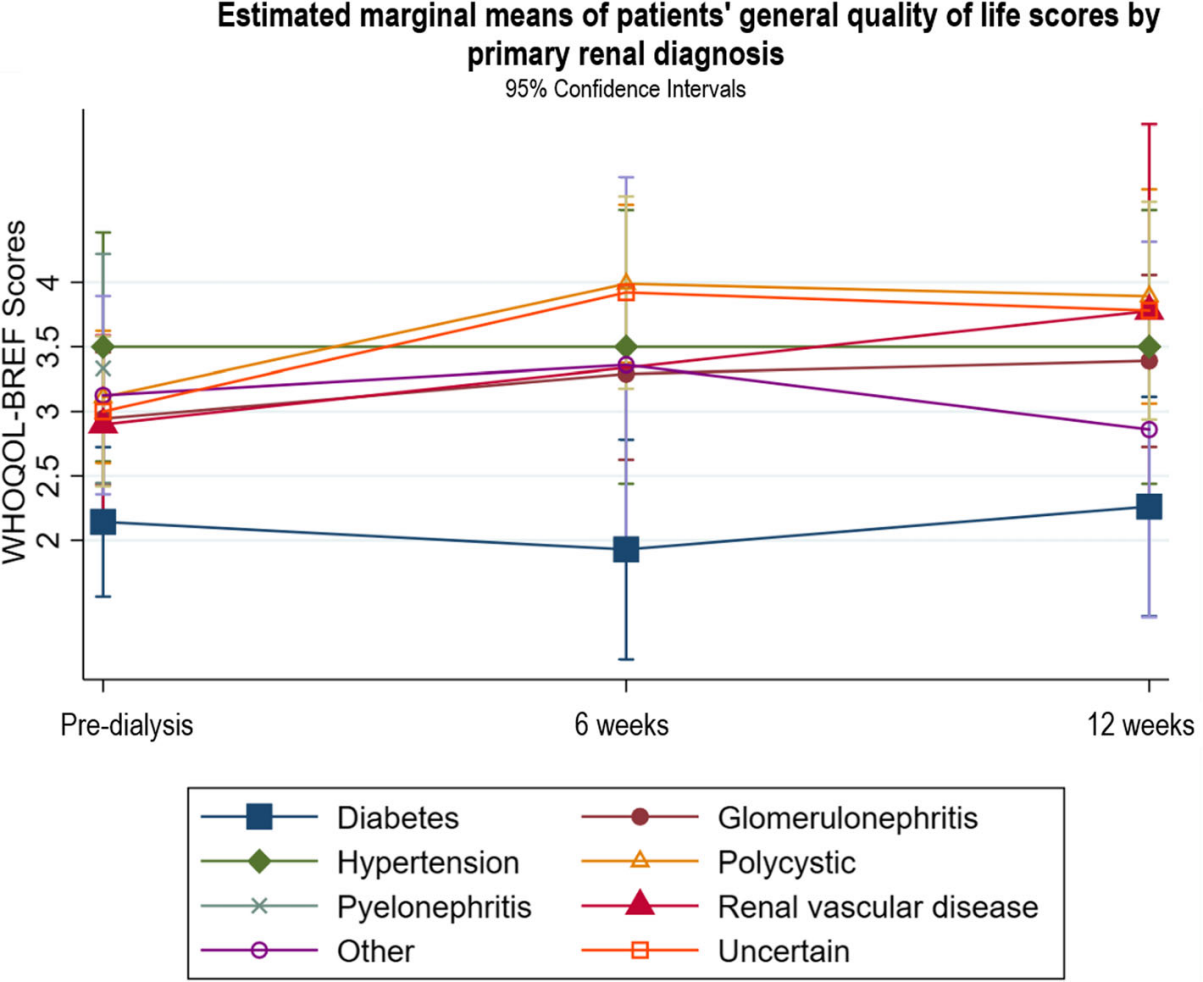
Estimated marginal means of patients’ general quality of life scores by primary renal diagnosis

**Fig. 9:**
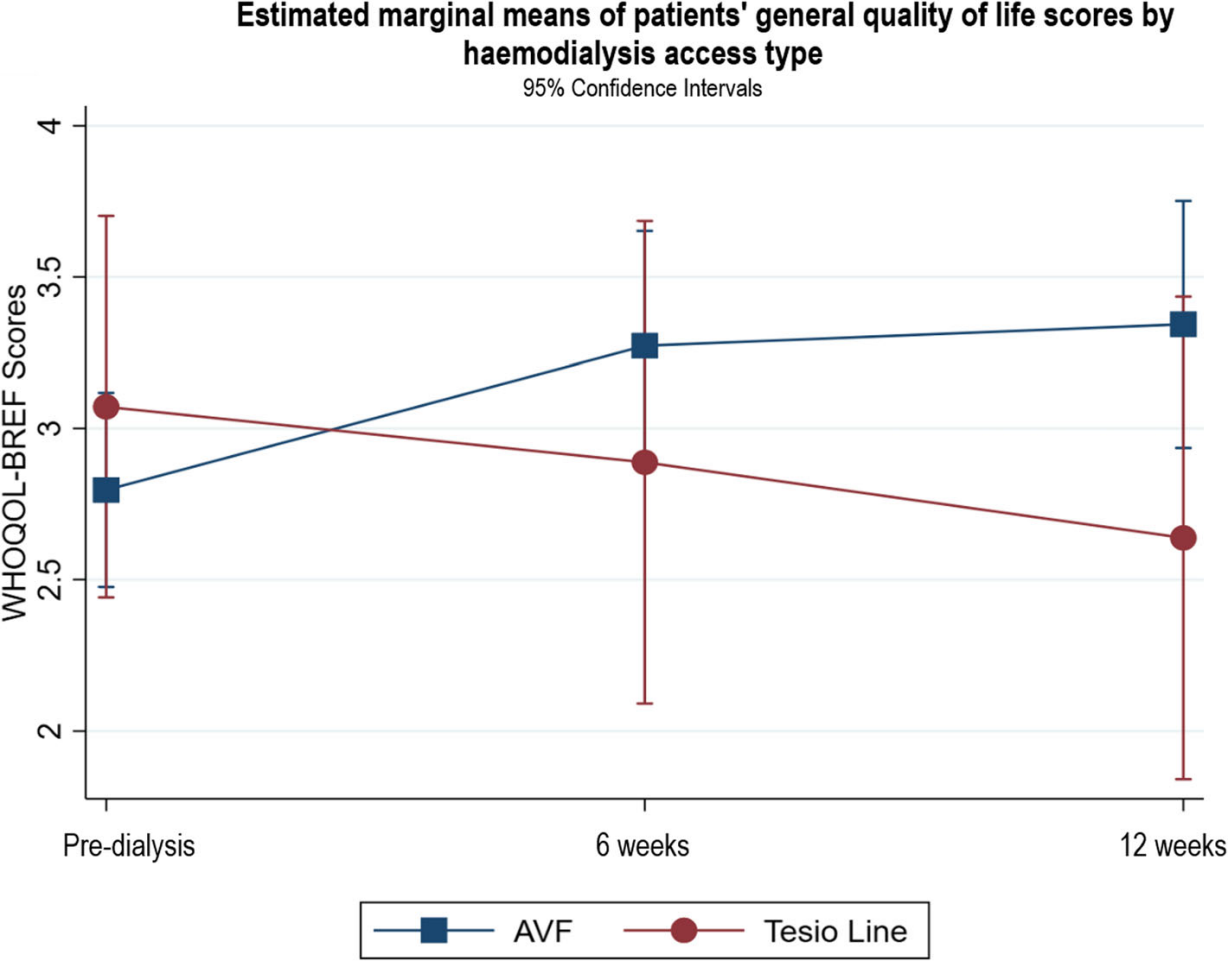
Estimated marginal means of patients’ general quality of life scores by haemodialysis access type

**Fig. 10:**
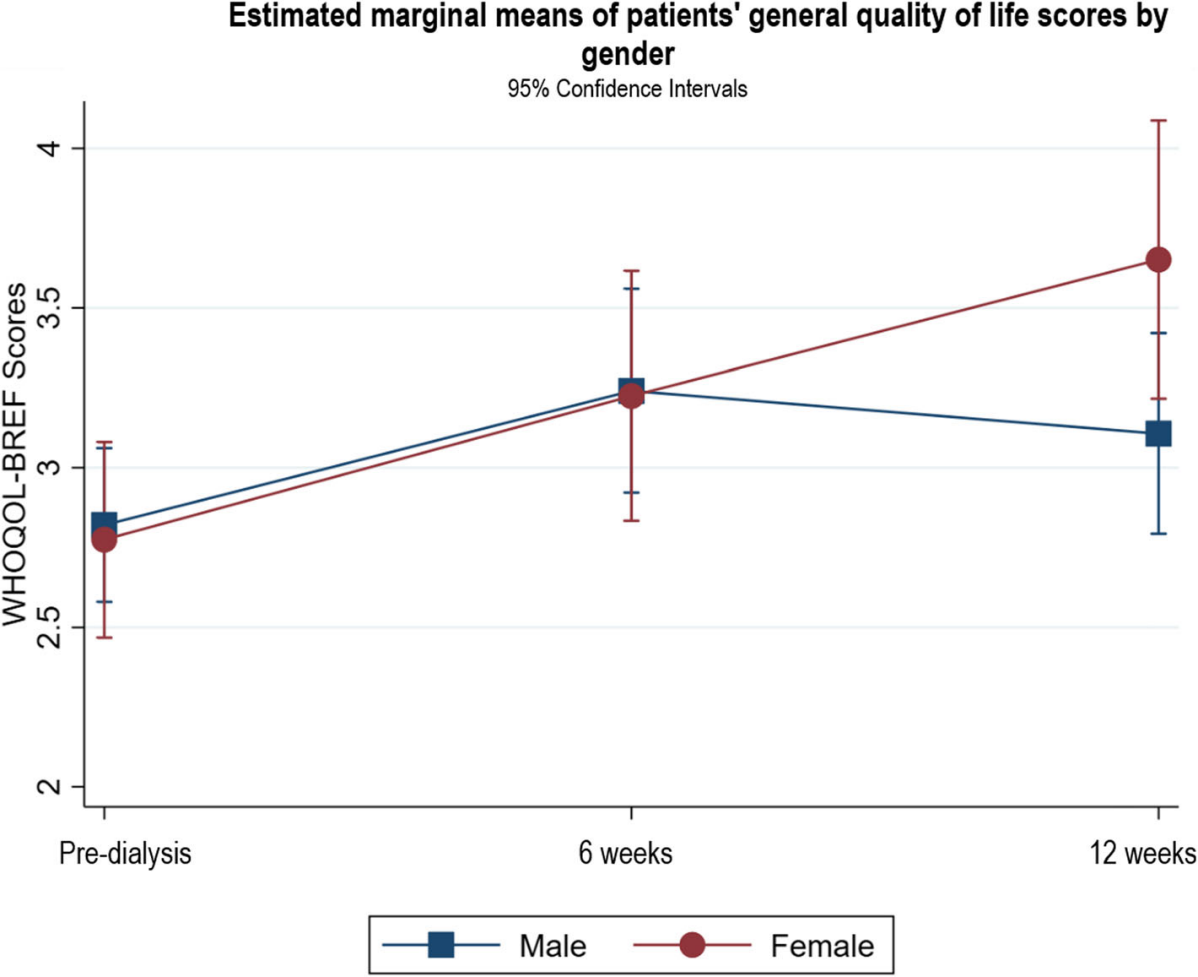
Estimated marginal means of patients’ general quality of life scores by gender

The correct figures have been included in this correction, and the original article has been corrected.
