# Supplementary material for: Quality of life improved for patients after starting dialysis but is impaired, initially, for their partners: a multi-centre, longitudinal study
Source: BMC Nephrol. 2020 May 18;21:185. doi: 10.1186/s12882-020-01819-4 (PMC7236460; doi:10.1186/s12882-020-01819-4)
Supplement: Supplementary file 1 — Additional file 1. Results of the multilevel models of changes in QOL in patients and partners and results from the linear comparison of parameters analysis in partners. [file 12882_2020_1819_MOESM1_ESM.docx]

**Supplementary Material**

| *Three level random intercept model of WHOQOL general quality of life between patients and partners* | | | |
| --- | --- | --- | --- |
|  | *Coefficients* | *95% CI* | *p value* |
| *Fixed effects* | | | |
| Patients’ general QOL | | | |
| Baseline (constant) | 2.8 | 2.62 - 2.99 |  |
| Patients change to 6 weeks | 0.42 | 0.19 - 0.65 | *p* <0.001 |
| Patients change to 12 weeks | 0.47 | 0.24 - 0.71 | *p* <0.001 |
| Partners’ general QOL relative to patients | | | |
| Baseline | 0.72 | 0.51 - 0.93 | *p* <0.001 |
| Partners change to 6 weeks | -0.66 | -0.98 - -0.34 | *p* < 0.001 |
| Partners change to 12 weeks | -0.56 | -0.89 - -0.23 | *p* < 0.001 |
|  |  |  |  |
| *Random effects* | *Estimate* | *SE* | *95% CI* |
| Between couple | 0.26 | 0.07 | 0.15 - 0.44 |
| Between participant | 0.13 | 0.05 | 0.06 – 0.28 |
| Note. CI=Confidence interval, QOL=quality of life, SE=Standard error, WHOQOL=World Health Organization QOL-BREF version. | | | |

**Additional File 1**

*Results of the linear combination of parameters analysis in partners’* *WHOQOL general QOL*

|  | *Coefficients* | *95% CI* | *p value* |
| --- | --- | --- | --- |
| Partners change to 6 weeks | -0.24 | -0.47 - -0.01 | *p* = 0.04 |
| Partners change to 12 weeks | -0.09 | -0.33 – 0.15 | *p* = 0.474 |
| Note. CI=Confidence interval, QOL=quality of life, WHOQOL=World Health Organization QOL-BREF version. | | | |

| *Three level random intercept model of WHOQOL physical domain between patients and partners* | | | |
| --- | --- | --- | --- |
|  | *Coefficients* | *95% CI* | *p value* |
| *Fixed effects* | | | |
| Patients’ physical domain | | | |
| Baseline (constant) | 46.02 | 41.28 – 50.76 |  |
| Patients change to 6 weeks | 3.44 | -0.97 - 7.85 | *p* = 0.126 |
| Patients change to 12 weeks | 6.56 | 2.10 - 11.03 | *p* = 0.004 |
| Partners’ physical domain relative to patients | | | |
| Baseline | 21.11 | 15.74 – 26.47 | *p* <0.001 |
| Partners change to 6 weeks | -5.02 | -11.13 – 1.09 | *p* = 0.107 |
| Partners change to 12 weeks | -7.58 | -13.83 - -1.33 | *p* 0.017 |
|  |  |  |  |
| *Random effects* | *Estimate* | *SE* | *95% CI* |
| Between couple | 165.85 | 50.73 | 91.06 – 302.06 |
| Between participant | 183.90 | 40.57 | 119.34 – 283.37 |
| Note. CI=Confidence interval, SE=Standard error, WHOQOL=World Health Organization QOL-BREF version. | | | |
| *Three level random intercept model of WHOQOL psychological domain between patients and partners* | | | |
|  | *Coefficients* | *95% CI* | *p value* |
| *Fixed effects* | | | |
| Patients’ psychological domain | | | |
| Baseline (constant) | 61.51 | 57.30 - 65.72 |  |
| Patients change to 6 weeks | -0.66 | -5.20 – 3.87 | *p* = 0.775 |
| Patients change to 12 weeks | -1.14 | -5.83 – 3.55 | *p* = 0.633 |
| Partners’ psychological domain relative to patients | | | |
| Baseline | 4.23 | -0.70 – 9.17 | *p* = 0.093 |
| Partners change to 6 weeks | -1.76 | -8.08 – 4.56 | *p* = 0.585 |
| Partners change to 12 weeks | -2.64 | -9.18 – 3.90 | *p* = 0.428 |
|  |  |  |  |
| *Random effects* | *Estimate* | *SE* | *95% CI* |
| Between couple | 116.17 | 37.41 | 61.79 – 218.41 |
| Between participant | 122.97 | 31.52 | 74.40 – 203.25 |
| Note. CI=Confidence interval, SE=Standard error, WHOQOL=World Health Organization QOL-BREF version. | | | |
| *Three level random intercept model of WHOQOL social domain between patients and partners* | | | |
|  | *Coefficients* | *95% CI* | *p value* |
| *Fixed effects* | | | |
| Patients’ social domain | | | |
| Baseline (constant) | 62.92 | 58.70 – 67.13 |  |
| Patients change to 6 weeks | 0.93 | -3.70 – 5.57 | *p* = 0.693 |
| Patients change to 12 weeks | 1.79 | -2.96 – 6.54 | *p* = 0.459 |
| Partners’ social domain relative to patients | | | |
| Baseline | 1.82 | -2.64 – 6.29 | *p* = 0.423 |
| Partners change to 6 weeks | -1.74 | -8.14- 4.67 | *p* = 0.595 |
| Partners change to 12 weeks | -3.29 | -9.88 – 3.31 | *p* = 0.329 |
|  |  |  |  |
| *Random effects* | *Estimate* | *SE* | *95% CI* |
| Between couple | 161.43 | 39.98 | 99.35 – 262.30 |
| Between participant | 71.66 | 23.90 | 37.27 – 137.78 |
| Note. CI=Confidence interval, SE=Standard error, WHOQOL=World Health Organization QOL-BREF version. | | | |
| *Three level random intercept model of WHOQOL environmental domain between patients and partners* | | | |
|  | *Coefficients* | *95% CI* | *p value* |
| *Fixed effects* | | | |
| Patients’ environment domain | | | |
| Baseline (constant) | 67.15 | 63.55 – 70.75 |  |
| Patients change to 6 weeks | -0.56 | -3.99 – 2.86 | *p* = 0.747 |
| Patients change to 12 weeks | -0.97 | -4.48 – 2.53 | *p* = 0.587 |
| Partners’ environment domain relative to patients | | | |
| Baseline | 0.47 | -3.17 – 4.10 | *p* = 0.802 |
| Partners change to 6 weeks | 0.68 | -4.05 – 5.41 | *p* = 0.778 |
| Partners change to 12 weeks | -1.65 | -6.51 – 3.22 | *p* = 0.507 |
|  |  |  |  |
| *Random effects* | *Estimate* | *SE* | *95% CI* |
| Between couple | 131.27 | 30.32 | 83.48 – 206.42 |
| Between participant | 64.90 | 17.20 | 38.60 – 109.10 |
| Note. CI=Confidence interval, SE=Standard error, WHOQOL=World Health Organization QOL-BREF version. | | | |
