# Supplementary material for: Quality of life improved for patients after starting dialysis but is impaired, initially, for their partners: a multi-centre, longitudinal study
Source: BMC Nephrol. 2020 May 18;21:185. doi: 10.1186/s12882-020-01819-4 (PMC7236460; doi:10.1186/s12882-020-01819-4)
Supplement: Supplementary file 2 — Additional file 2. Results of the multilevel models of changes in QOL scores of patients and partners in relation to baseline clinical and socio-demographic variables. [file 12882_2020_1819_MOESM2_ESM.docx]

**Supplementary Material**

**Additional File 2**

| *Two level random intercept model of WHOQOL general quality of life in patients by comorbidity risk* | | | |
| --- | --- | --- | --- |
|  | *Coefficients* | *95% CI* | *p value* |
| *Fixed effects* | | | |
| Low risk patients’ general QOL | | | |
| Baseline (constant) | 3.0 | 2.67 - 3.33 |  |
| Patients change to 6 weeks | 0.38 | -0.08 – 0.83 | *p* = 0.104 |
| Patients change to 12 weeks | 0.58 | 0.11 – 1.04 | *p* = 0.016 |
| Medium risk compared to low risk patients | | | |
| Baseline | 0.06 | -0.36 – 0.48 | *p* = 0.770 |
| MRP change to 6 weeks | -0.13 | -0.69 – 0.43 | *p* = 0.656 |
| MRP change to 12 weeks | -0.41 | -0.98 – 0.17 | *p* = 0.166 |
|  |  |  |  |
| High risk compared to low risk patients | |  |  |
| Baseline | -1.0 | -1.61 - -0.39 | *p* = 0.001 |
| HRP change to 6 weeks | 0.89 | 0.01 – 1.78 | *p* = 0.048 |
| HRP change to 12 weeks | 0.35 | -0.55 – 1.24 | *p* = 0.448 |
|  |  |  |  |
| *Random effects* | *Estimate* | *SE* | *95% CI* |
| Between participant | 0.30 | 0.09 | 0.17 – 0.53 |
| Note. CI=Confidence interval, HRP=High risk patients, MRP=Medium risk patients, SE=Standard error, QOL=Quality of life, WHOQOL=World Health Organization QOL-BREF version. Comorbidity risk levels were determined following the guidelines set out by Davies et al. ([25](#_ENREF_25)). | | | |

| *Two level random intercept model of WHOQOL general quality of life in patients by primary renal diagnosis* | | | |
| --- | --- | --- | --- |
|  | *Coefficients* | *95% CI* | *p value* |
| *Fixed effects* | | | |
| General QOL in patients with PRD: Diabetes | | | |
| Baseline (constant) | 2.14 | 1.56 – 2.72 |  |
| Change to 6 weeks | -0.21 | -1.11 – 0.69 | *p* = 0.642 |
| Change to 12 weeks | 0.12 | 0.78 – 1.02 | *p* = 0.794 |
| Patients PRD: Glomerulonephritis compared to patients with PRD: Diabetes | | | |
| Baseline | 0.80 | 0.03 – 1.58 | *p* = 0.042 |
| Change to 6 weeks | 0.56 | -0.59 – 1.71 | *p* = 0.340 |
| Change to 12 weeks | 0.33 | -0.82 – 1.48 | *p* = 0.578 |
| Patients PRD: Hypertension compared to patients with PRD: Diabetes | | |  |
| Baseline | 1.36 | 0.30 – 2.42 | *p* = 0.012 |
| Change to 6 weeks | 0.21 | -1.25 – 1.68 | *p* = 0.775 |
| Change to 12 weeks | -0.12 | -1.58 – 1.34 | *p* = 0.873 |
| Patients PRD: Polycystic compared to patients with PRD: Diabetes | | |  |
| Baseline | 0.97 | 0.19 – 1.74 | *p* = 0.014 |
| Change to 6 weeks | 1.09 | -0.03 – 2.21 | *p* = 0.056 |
| Change to 12 weeks | 0.66 | -0.59 – 1.91 | *p* = 0.301 |
| Patients PRD: Pyelonephritis compared to patients with PRD: Diabetes | | |  |
| Baseline | 1.19 | 0.13 – 2.25 | *p* = 0.028 |
| Change to 6 weeks | - | - | *-* |
| Change to 12 weeks | - | - | *-* |
| Patients PRD: Renal vascular disease compared to patients with PRD: Diabetes | | |  |
| Baseline | 0.76 | -0.14 – 1.66 | *p* = 0.099 |
| Change to 6 weeks | - | - | *-* |
| Change to 12 weeks | 0.76 | -0.98 – 2.50 | *p* = 0.394 |
| Patients PRD: Other compared to patients with PRD: Diabetes | | |  |
| Baseline | 0. 98 | 0.02 – 1.94 | *p* = 0.046 |
| Change to 6 weeks | 0.45 | -1.31 – 2.20 | *p* = 0.616 |
| Change to 12 weeks | -0.38 | -2.14 – 1.37 | *p* = 0.668 |
| Patients PRD: Unknown compared to patients with PRD: Diabetes | | |  |
| Baseline | 0.86 | 0.04 – 1.68 | *p* = 0.041 |
| Change to 6 weeks | 1.14 | -0.07 – 2.34 | *p* = 0.065 |
| Change to 12 weeks | 0.66 | -0.61 – 1.93 | *p* = 0.307 |
|  |  |  |  |
| *Random effects* | *Estimate* | *SE* | *95% CI* |
| Between participant | 0.23 | 0.12 | 0.01 – 0.62 |
| Note. CI=Confidence interval, PRD=Primary Renal Diagnosis, QOL=Quality of life, SE=Standard error, WHOQOL=World Health Organization QOL-BREF version. | | | |
| *Two level random intercept model of WHOQOL general quality of life in patients by haemodialysis access type* | | | |
|  | *Coefficients* | *95% CI* | *p value* |
| *Fixed effects* | | | |
| General QOL in patients with AVF | | | |
| Baseline (constant) | 2.79 | 2.48 – 3.12 |  |
| Change to 6 weeks | 0.48 | 0.08 – 0.87 | *p* = 0.018 |
| Change to 12 weeks | 0.55 | 0.13 – 0.97 | *p* = 0.011 |
| Patients with tesio line compared to patients with AVF | | | |
| Baseline | 0.28 | -0.43 – 0.98 | *p* = 0.446 |
| Change to 6 weeks | -0.66 | -1.57 – 0.25 | *p* = 0.156 |
| Change to 12 weeks | -0.98 | -1.90 – -0.06 | *p* = 0.037 |
|  |  |  |  |
|  |  |  |  |
| *Random effects* | *Estimate* | *SE* | *95% CI* |
| Between participant | 0.33 | 0.16 | 0.12 – 0.88 |
| Note. AVF=arteriovenous fistula, CI=Confidence interval, QOL=Quality of life, SE=Standard error, WHOQOL=World Health Organization QOL-BREF version. | | | |
| *Two level random intercept model of WHOQOL general quality of life in patients by gender* | | | |
|  | *Coefficients* | *95% CI* | *p value* |
| *Fixed effects* | | | |
| Male patients’ general QOL | | | |
| Baseline (constant) | 2.82 | 2.58 – 3.06 |  |
| Patients change to 6 weeks | 0.42 | 0.1 – 0.74 | *p* = 0.011 |
| Patients change to 12 weeks | 0.29 | -0.03 – 0.60 | *p* = 0.077 |
| Female patients compared to male patients | | | |
| Baseline | -0.05 | -0.44 – 0.34 | *p* = 0.818 |
| FP change to 6 weeks | 0.03 | -0.48 – 0.54 | *p* = 0.910 |
| FP change to 12 weeks | 0.60 | 0.05 – 1.13 | *p* = 0.033 |
|  |  |  |  |
|  |  |  |  |
| *Random effects* | *Estimate* | *SE* | *95% CI* |
| Between participant | 0.36 | 0.1 | 0.21 – 0.62 |
| Note. CI=Confidence interval, FP=Female patients, QOL=Quality of life, SE=Standard error, WHOQOL=World Health Organization QOL-BREF version. | | | |

| *Two level random intercept model of WHOQOL general quality of life in patients in relation to baseline haemoglobin* | | | |
| --- | --- | --- | --- |
|  | *Coefficients* | *95% CI* | *p value* |
| *Fixed effects* | | | |
| General QOL | | | |
| Baseline (constant) | 2.40 | 1.06 – 3.73 |  |
| Change to 6 weeks | 0.01 | -0.01 – 0.03 | *p* = 0.211 |
| Change to 12 weeks | 0.01 | -0.01 – 0.03 | *p* = 0.266 |
|  |  |  |  |
| *Random effects* | *Estimate* | *SE* | *95% CI* |
| Between participant | 0.34 | 0.10 | 0.12 – 0.60 |
| Note. CI=Confidence interval, QOL=Quality of life, SE=Standard error, WHOQOL=World Health Organization QOL-BREF version. | | | |

| *Two level random intercept model of WHOQOL general quality of life in patients in relation to baseline serum albumin* | | | |
| --- | --- | --- | --- |
|  | *Coefficients* | *95% CI* | *p value* |
| *Fixed effects* | | | |
| General QOL | | | |
| Baseline (constant) | 1.87 | 0.63 – 3.11 |  |
| Change to 6 weeks | 0.03 | -0.02 – 0.07 | *p* = 0.294 |
| Change to 12 weeks | 0.00 | -0.05 – 0.05 | *p* = 0.904 |
|  |  |  |  |
| *Random effects* | *Estimate* | *SE* | *95% CI* |
| Between participant | 0.31 | 0.01 | 0.17 – 0.56 |
| Note. CI=Confidence interval, QOL=Quality of life, SE=Standard error, WHOQOL=World Health Organization QOL-BREF version. | | | |

| *Two level random intercept model of WHOQOL general quality of life in patients in relation to baseline eGFR* | | | |
| --- | --- | --- | --- |
|  | *Coefficients* | *95% CI* | *p value* |
| *Fixed effects* | | | |
| General QOL | | | |
| Baseline (constant) | 3.28 | 0.63 – 3.11 |  |
| Change to 6 weeks | -0.06 | -0.18 – 0.05 | *p* = 0.281 |
| Change to 12 weeks | -0.03 | -0.14 – 0.09 | *p* = 0.644 |
|  |  |  |  |
| *Random effects* | *Estimate* | *SE* | *95% CI* |
| Between participant | 0.32 | 0.10 | 0.18 – 0.57 |
| Note. CI=Confidence interval, eGFR=estimated glomerular filtration rate, QOL=Quality of life, SE=Standard error, WHOQOL=World Health Organization QOL-BREF version. | | | |
| *Two level random intercept model of WHOQOL general quality of life in patients in relation to baseline age* | | | |
|  | *Coefficients* | *95% CI* | *p value* |
| *Fixed effects* | | | |
| General QOL | | | |
| Baseline (constant) | 2.49 | 1.60 – 3.38 |  |
| Change to 6 weeks | 0.00 | -0.02 – 0.02 | *p* = 0.688 |
| Change to 12 weeks | 0.01 | -0.01 – 0.03 | *p* = 0.458 |
|  |  |  |  |
| *Random effects* | *Estimate* | *SE* | *95% CI* |
| Between participant | 0.34 | 0.10 | 0.20 – 0.60 |
| Note. CI=Confidence interval, QOL=Quality of life, SE=Standard error, WHOQOL=World Health Organization QOL-BREF version. | | | |

| *Two level random intercept model of WHOQOL general quality of life in patients by patient type* | | | |
| --- | --- | --- | --- |
|  | *Coefficients* | *95% CI* | *p value* |
| *Fixed effects* | | | |
| General QOL in incident patients | | | |
| Baseline (constant) | 2.78 | 2.55 – 3.00 |  |
| Change to 6 weeks | 0.43 | 0.12 – 0.75 | *p* = 0.007 |
| Change to 12 weeks | 0.40 | 0.08 – 0.72 | *p* = 0.014 |
| Prevalent patients’ changes in QOL compared to incident patients | | | |
| Baseline | 0.30 | -0.39 – 1.00 | *p* = 0.391 |
| Change to 6 weeks | 0.12 | -0.88 – 0.90 | *p* = 0.978 |
| Change to 12 weeks | 0.17 | -0.72 – 1.06 | *p* = 0.706 |
|  |  |  |  |
|  |  |  |  |
| *Random effects* | *Estimate* | *SE* | *95% CI* |
| Between participant | 0.28 | 0.11 | 0.13 – 0.58 |
| Note. CI=Confidence interval, QOL=Quality of life, SE=Standard error, WHOQOL=World Health Organization QOL-BREF version. Incident patient means a patient starting dialysis for the first time; prevalent refers to a patient who has been on a form of renal replacement therapy before but who intends to start dialysis due to a failing transplant. | | | |

| *Two level random intercept model of WHOQOL general quality of life in patients by mode of dialysis* | | | |
| --- | --- | --- | --- |
|  | *Coefficients* | *95% CI* | *p value* |
| *Fixed effects* | | | |
| General QOL in HD patients | | | |
| Baseline (constant) | 2.91 | 2.67 – 3.15 |  |
| Change to 6 weeks | 0.48 | 0.15 – 0.82 | *p* = 0.004 |
| Change to 12 weeks | 0.41 | 0.06 – 0.77 | *p* = 0.022 |
| PD patients’ changes in QOL compared to HD patients | | | |
| Baseline | -0.10 | -0.53 – 0.32 | *p* = 0.632 |
| Change to 6 weeks | -0.14 | -0.81 – 0.35 | *p* = 0.432 |
| Change to 12 weeks | 0.08 | -0.68 – 0.47 | *p* = 0.725 |
|  |  |  |  |
|  |  |  |  |
| *Random effects* | *Estimate* | *SE* | *95% CI* |
| Between participant | 0.32 | 0.10 | 0.17 – 0.59 |
| Note. CI=Confidence interval, HD=haemodialysis, PD=peritoneal dialysis, QOL=Quality of life, SE=Standard error, WHOQOL=World Health Organization QOL-BREF version. | | | |

| *Two level random intercept model of WHOQOL general quality of life in patients by start of dialysis* | | | |
| --- | --- | --- | --- |
|  | *Coefficients* | *95% CI* | *p value* |
| *Fixed effects* | | | |
| General QOL in patients with planned start | | | |
| Baseline (constant) | 2.88 | 2.64 – 3.11 |  |
| Change to 6 weeks | 0.37 | 0.07 – 0.66 | *p* = 0.016 |
| Change to 12 weeks | 0.44 | 0.13 – 0.74 | *p* = 0.005 |
| Changes in QOL in patients with unplanned started compared to patients with planned start | | | |
| Baseline | 0.13 | -0.76 – 1.01 | *p* = 0.782 |
| Change to 6 weeks | -0.53 | -1.56 – 0.49 | *p* = 0. 309 |
| Change to 12 weeks | -0.94 | -1.96 – 0.09 | *p* = 0.074 |
|  |  |  |  |
|  |  |  |  |
| *Random effects* | *Estimate* | *SE* | *95% CI* |
| Between participant | 0.36 | 0.11 | 0.19 – 0.66 |
| Note. CI=Confidence interval, QOL=Quality of life, SE=Standard error, WHOQOL=World Health Organization QOL-BREF version. | | | |

| *Two level random intercept model of WHOQOL general quality of life in partners in relation to baseline age* | | | |
| --- | --- | --- | --- |
|  | *Coefficients* | *95% CI* | *p value* |
| *Fixed effects* | | | |
| General QOL | | | |
| Baseline (constant) | 3.57 | 3.35 – 3.79 |  |
| Change to 6 weeks | -0.01 | -0.02 – 0.00 | *p* = 0.168 |
| Change to 12 weeks | -0.01 | -0.02 – 0.00 | *p* = 0.143 |
|  |  |  |  |
| *Random effects* | *Estimate* | *SE* | *95% CI* |
| Between participant | 0.41 | 0.09 | 0.27 – 0.64 |
| Note. CI=Confidence interval, QOL=Quality of life, SE=Standard error, WHOQOL=World Health Organization QOL-BREF version. | | | |

| *Two level random intercept model of WHOQOL general quality of life in partners by gender* | | | |
| --- | --- | --- | --- |
|  | *Coefficients* | *95% CI* | *p value* |
| *Fixed effects* | | | |
| Male partners’ general QOL | | | |
| Baseline (constant) | 3.52 | 3.22 – 3.81 |  |
| MP change to 6 weeks | -0.22 | -0.56 – 0.11 | *p* = 0.184 |
| MP change to 12 weeks | 0.10 | -0.26 – 0.46 | *p* = 0.585 |
| Female partners compared to male patients | | | |
| Baseline | 0.02 | -0.34 – 0.39 | *p* = 0.902 |
| FP change to 6 weeks | -0.05 | -0.48 – 0.37 | *p* = 0.799 |
| FP change to 12 weeks | -0.30 | -0.75 – 0.14 | *p* = 0.178 |
|  |  |  |  |
|  |  |  |  |
| *Random effects* | *Estimate* | *SE* | *95% CI* |
| Between participant | 0.36 | 0.1 | 0.21 – 0.62 |
| Note. CI=Confidence interval, FP=Female partners, MP=Male partners, QOL=Quality of life, SE=Standard error, WHOQOL=World Health Organization QOL-BREF version. | | | |
